# Comparing the Effects of Combined Oral Contraceptives Containing Progestins With Low Androgenic and Antiandrogenic Activities on the Hypothalamic-Pituitary-Gonadal Axis in Patients With Polycystic Ovary Syndrome: Systematic Review and Meta-Analysis

**DOI:** 10.2196/resprot.9024

**Published:** 2018-04-25

**Authors:** Mina Amiri, Fahimeh Ramezani Tehrani, Fatemeh Nahidi, Ali Kabir, Fereidoun Azizi

**Affiliations:** ^1^ Students Research Committee School of Nursing and Midwifery, Department of Midwifery and Reproductive Health Shahid Beheshti University of Medical Sciences Tehran Islamic Republic Of Iran; ^2^ Reproductive Endocrinology Research Center Research Institute for Endocrine Sciences Shahid Beheshti University of Medical Sciences Tehran Islamic Republic Of Iran; ^3^ School of Nursing and Midwifery Department of Midwifery and Reproductive Health Shahid Beheshti University of Medical Sciences Tehran Islamic Republic Of Iran; ^4^ Minimally Invasive Surgery Research Center Iran University of Medical Sciences Tehran Islamic Republic Of Iran; ^5^ Endocrine Research Center Shahid Beheshti University of Medical Sciences Tehran Islamic Republic Of Iran

**Keywords:** meta-analysis, combined oral contraceptives, androgens, gonadotropins, polycystic ovary syndrome

## Abstract

**Background:**

Different products of combined oral contraceptives (COCs) can improve clinical and biochemical findings in patients with polycystic ovary syndrome (PCOS) through suppression of the hypothalamic-pituitary-gonadal (HPG) axis.

**Objective:**

This systematic review and meta-analysis aimed to compare the effects of COCs containing progestins with low androgenic and antiandrogenic activities on the HPG axis in patients with PCOS.

**Methods:**

We searched PubMed, Scopus, Google Scholar, ScienceDirect, and Web of Science databases (1980-2017) to identify randomized controlled trials or nonrandomized studies investigating the effect of COCs containing progestins with low androgenic and antiandrogenic activities, including the products containing desogestrel, cyproterone acetate, and drospirenone, on the HPG axis in patients with PCOS. In this meta-analysis, fixed and random effect models were used. Outcomes of interest were weighted mean differences (WMD) of hormonal parameters, including the follicle-stimulating hormone (FSH), luteinizing hormone (LH), LH-to-FSH ratio, estradiol, total testosterone, and sex hormone–binding globulin. Potential sources of heterogeneity were investigated using meta-regression and subgroup analyses. Subgroup analyses were performed based on the used progestin compound and treatment duration. We assessed quality of included studies and their risk of bias using Cochrane guidelines. Publication bias was assessed using Egger test and funnel plot.

**Results:**

COC use was significantly associated with a decrease in gonadotropin levels, including FSH and LH. Use of products containing cyproterone acetate was associated with a decrease in FSH levels after 3 months (WMD=−0.48; 95% CI −0.81 to −0.15), 6 months (WMD=−2.33; 95% CI −3.48 to −1.18), and 12 months (WMD=−4.70; 95% CI −4.98 to −4.42) and a decrease in LH levels after 3 months (WMD=−3.57; 95% CI −5.14 to −1.99), 6 months (WMD=−5.68; 95% CI −9.57 to −1.80), and 12 months (WMD=−11.60; 95% CI −17.60 to −5.60). Use of COCs containing drospirenone for 6 months decreased FSH (WMD=−0.93; 95% CI −1.79 to −0.08) and LH (WMD=−4.59; 95% CI −7.53 to −1.66) levels. Data for products containing desogestrel were few, but this compound generally had no statistically significant influence on gonadotropin levels similar to that observed with COCs containing cyproterone acetate and drospirenone. Use of COCs was not associated with any significant change in LH-to-FSH ratio. COCs containing cyproterone acetate showed maximum effect on gonadotropin suppression. COCs containing cyproterone acetate significantly decreased estradiol concentrations, whereas those containing drospirenone exhibited no such effect. All COCs demonstrated improvement in androgenic profile and had the same effects on total testosterone and sex hormone–binding globulin concentrations. Progestin compound and treatment duration had no statistically significant effects on changing total testosterone and sex hormone–binding globulin levels.

**Conclusions:**

COCs containing cyproterone acetate can effectively suppress gonadotropins, leading to a decrease in androgenic parameters. Although different products of COCs could significantly suppress the androgenic profile, it seems that products containing cyproterone acetate are more effective in suppressing gonadotropin and estradiol levels in patients with PCOS.

## Introduction

Polycystic ovary syndrome (PCOS) is a common endocrine and metabolic disorder in reproductive age women [1-3], characterized by chronic oligo and/anovulation and hyperandrogenism (HA), which results in infertility, menstrual irregularities, hirsutism, acne, and alopecia [4]. PCOS is associated with an increase in risk of metabolic disorders such as obesity, dyslipidemia, and impaired glucose metabolism, which in turn increase the risk of diabetes mellitus and cardiovascular disease [3,5,6]. This endocrine disorder can have negative effects on the health-related quality of life of these women [7].

Combined oral contraceptives (COCs) are considered as the most common symptomatic treatment of PCOS and contain a combination of estrogen and progestin [8]. COCs are used not only to regulate menstrual cycle but also to suppress the hypothalamic-pituitary-gonadal (HPG) axis and improve clinical and biochemical HA in women with PCOS [9].

The effectiveness of COCs for the treatment of PCOS is well documented [10]. Previous studies show that COCs affect androgen synthesis by inhibiting ovarian androgen production [11-13]. The main potential mechanisms of COC action include inhibition of folliculogenesis as a result of suppression of gonadotropin secretion, suppression of ovarian and adrenal androgen synthesis, inhibition of 5 alpha reductase, and increased sex hormone–binding globulin (SHBG) [14,15]. Hence, COCs can improve the HPG axis function through a decrease in gonadotropin and ovarian androgen levels, which is a major goal of PCOS treatment [16].

Progestin activity of COCs inhibits luteinizing hormone (LH) secretion and results in a decline in ovarian androgen release [17]. Current COC products containing newer progestins with low androgenic or antiandrogenic effects, such as cyproterone acetate (CA), chlormadinone acetate (CMA), desogestrel (DSG), and drospirenone (DRSP), are considered to be effective in decreasing gonadotropin and androgen levels [14,18,19]. In particular, these progestins are better for women with PCOS suffering from HA [17].

Although the effect of COCs on the HPG axis of PCOS women has been introduced before, however, to the best of our knowledge, there is no other meta-analysis comparing this effect among COCs with various progesterone components. In our opinion, this is a valuable piece of knowledge that could provide some clues for a better understanding of the mechanism of effect of various COC compounds, which may be helpful in the decision-making process for treatment options.

This meta-analysis aimed to compare the effects of COCs containing progestins with low androgenic and antiandrogenic activities on the HPG axis in patients with PCOS.

## Methods

### Overview

This systematic review and meta-analysis was designed according to the PRISMA (Preferred Reporting Items for Systematic Reviews and Meta-Analyses) statement (Multimedia Appendix 1) [20] and the Cochrane Handbook for Systematic Reviews of Interventions [21] to answer the following questions:

1. Do COCs affect the HPG axis of women with PCOS?
2. Is there any difference among the effects of COCs on the HPG axis in women with PCOS?
3. Is there any difference in the effects of these compounds based on the duration of their use?

The study was approved by the ethics committee of Research Institute for Endocrine Sciences, Shahid Beheshti University of Medical Sciences, Tehran, Iran (Registration number: IR.SBMU.RIES.RES.1394.90).

### Search Strategy

PubMed, Scopus, Google Scholar, ScienceDirect, and Web of Science were searched for clinical trials investigating the influence of COCs containing progestins with low androgenic and antiandrogenic activities on the HPG axis in patients with PCOS from January 1980 to June 2017. After searching for subheadings of PCOS in MeSH, the following keyword combinations were selected: [“polycystic ovary syndrome” AND “contracept^*^”] and [“polycystic ovary syndrome” AND “contraceptives, oral hormonal” OR “pill” OR “progestin”]. Search limitations were human, females, clinical trial, and English language.

A hand search of the reference lists of all selected papers was also conducted to prevent missing studies.

### Eligibility Criteria

Studies conducted on reproductive age women with PCOS who were treated with monophasic COCs were selected for this meta-analysis; these studies were randomized clinical trials (RCTs) or nonrandomized studies (NRS).

Diagnostic criteria of each study are identified in Multimedia Appendix 2. In all the included studies, nonclassic congenital adrenal hyperplasia, hyperprolactynemia, and other HA etiologies were ruled out. The intervention of interest was COC containing progestins with low androgenic or antiandrogenic activities. Follicle-stimulating hormone (FSH), LH, LH-to-FSH ratio, estradiol (E2), total testosterone (TT), and SHBG levels were considered as main outcomes of the study.

Exclusion criteria were as follows: (1) women with idiopathic hirsutism or other types of HA, (2) women with diabetes or other chronic diseases, (3) use of biphasic or triphasic contraceptives, (4) use of gonadotropin-releasing hormone agonist-antagonist and antiandrogen drugs (eg, ketoconazole and spironolactone), (5) studies with follow-ups of <3 months or >24 months, (6) use of biphasic and triphasic COCs, (7) use of progesterone-only compounds, (8) use of metformin in combination with COC, and (9) treatment groups with inadequate number of participants for performing meta-analysis (<1 study).

Only one study had a follow-up of 24 months and was excluded from the analysis [9]. We also excluded intervention groups that had no adequate number of study participants for performing a meta-analysis, including products containing levonorgestrel (LNG) and gestodene (GSD). In addition, study groups that assessed metformin + COCs were excluded from the analysis.

### Study Selection

We included all relevant RCTs or NRS assessing COC effects on the HPG axis in reproductive age women with PCOS. At least one of the following hormonal parameters had to be reported: FSH, LH, LH-to-FSH ratio, E2, TT, or SHBG. We considered COCs containing CPA, DRSP, DSG, and CMA as interventions of interest. Due to inadequate number of studies that assessed products with LNG and DSG, these were excluded from the study.

The results of the searches were screened for meeting the predefined eligibility criteria. All references were entered to the endnote software. Selection was performed based on their titles, followed by using a second selection performed by 1 reviewer (MA), who deleted duplicates and reviewed abstracts of all remaining records. Any disagreement in the selection of abstracts was resolved by consensus or by another reviewer (FRT). Full-text articles for review and data processing were obtained for all selected abstracts.

### Data Extraction

For each study, the following information were extracted: authors, year of publication, title, study design, characteristics of study population, type of intervention, outcome measurements—including FSH, milliunits per milliliters (mU/mL); LH, mU/mL; E2, picograms per milliliter; TT, nanograms per milliliter; and SHBG, nanomole per liter—and analytical methods. After data extraction, all the measurement units of hormones were identical. Data were extracted from full-text articles by 2 reviewers (MA and AK) in close consultation with another reviewer (FRT).

Data of studies were extracted by mean and SD [22]. To prevent extraction errors, a control check between the final data used in the meta-analysis and the original publications was performed by all authors.

### Quality Assessment

Two reviewers (MA and AK) assessed the quality of the studies separately. They were blinded to study author, institution, and journal name. Disagreement was resolved and adjusted by the senior reviewer (FRT). A validated quality assessment checklist for clinical trial as the modified Consolidated Standards of Reporting Trials (CONSORT) was used to assign a score to each paper. The quality assessment of RCTs was assessed based on the 37-item CONSORT checklist. Each of the 37 items included in CONSORT were scored to compute an overall quality score (range 0-37). For scoring of the quality of items, 1 point was given if the information for each item was stated in the study, and 0 was given if the information was not stated or was unclear. CONSORT was also modified to the NRS, which were not randomized controlled studies. For modification of the checklist, questions related to the blinding and randomization were excluded.

All clinical trial papers were categorized into 4 groups: high, moderate, low, and very low quality. Studies with scores ≥70% of the highest level of the CONSORT checklist were considered as high, 40% to 70% as moderate, 20% to 40% as low, and <20% as very low quality [23].

### Risk of Bias Assessment

Two authors (MA and AK) independently assessed risk of bias. The risk of bias in each included study was assessed using the criteria outlined in the Cochrane Handbook for Systematic Reviews of Interventions [21,24,25]. Six domains related to risk of bias were assessed in each included RCTs: (1) random sequence generation; (2) allocation concealment; (3) blinding of participants and personnel; (4) blinding of outcome assessment; (5) incomplete outcome data; and (6) selective reporting. Review authors’ judgments were categorized as “low risk,” “high risk,” and “unclear risk” of bias [24].

For NRS, 7 domains were assessed, including (1) confounding, (2) enrollment of participants in the study, (3) classification of interventions, (4) deviations from intended, (5) missing data, (6) measurement of outcomes, and (7) selection of the reported results. Review authors’ judgments were categorized as “low risk,” “moderate risk,” “serious risk,” “critical or high risk,” and “unclear or no information risk” of bias [25].

We planned to assess outcomes based on the risk of bias in the following subgroups: (1) low risk, (2) moderate risk, (3) serious/high/critical risk, and (4) unclear or no information risk.

### Statistical Methods

The studies selected assessed the effects of one or more COCs. Means and SDs of data at baseline and after treatment were collected. For studies reporting median and range, a conversion to mean and SD was performed, when possible [26]. Differences of mean and SD at both baseline and at end of treatment were calculated, as were standard errors of these differences, using the *Cochrane Reviewers’ Handbook*. For effect measures, the mean difference [27] and related 95% CIs were calculated based on the means of the pretreatment and those at the end of treatment levels of FSH, LH, LH-to-FSH ratio, E2, TT, and SHBG. Therefore, the primary pooled effect analysis was estimated weighted mean differences (WMD) for the studies comparing treatment groups of studies. For the end-of-treatment time point, the assessment (mean/SD) after a 3-, 6-, or 12-month cycle was used [21].

Heterogeneity tests were assessed by I-squared and chi-squared tests [28]. Both fixed and random effect models were used in the study. The random effect estimation method was applied for significant chi-squared test results (*P*<.10) or I-squared greater than 50%.

Subgroup analyses were performed based on COC compound and duration of use. In addition to funnel plot, Begg test [27] and Egger test [29] were used to assess publication bias. Publication bias was found to be significant for *P* values <.10 to indicate significant asymmetry. For significant results or asymmetric funnel plot, the trim and fill method (by metatrim) was used to identify and correct for publication bias. Metatrim is a command used in the STATA version 12 software (StataCorp, College Station, TX, USA) for overcoming publication bias. It simulates studies that have not been published in literature and assesses whether the results would be different when there is or there is no publication bias. Indeed, for significant results or asymmetric funnel plot, the trim and fill method (by metatrim) was used to identify and correct for publication bias by adding some study measures [30,31]. We used meta-regression to evaluate heterogeneity induced by important variables, including diagnostic criteria of PCO (Rotterdam; National Institutes of Health, NIH; Androgen Excess Society, AES]; and others), body mass index (BMI), and method of assay of different hormones (radioimmunoassay, chemical/electrochemical luminescence, enzyme, and unknown). In addition, we used metainf for performing the sensitivity analysis. We also assessed risk of bias for included studies using the Risk of Bias tools as per the Cochrane guidelines, which are tools designed for RCTs and NRS [24,25]. We then performed a subgroup analysis based on the risk of bias. *P* values <.05 were considered significant for all comparisons, except for heterogeneity, publication bias, and meta-regression, where .10 was set as the significance level. All analyses were performed with STATA software, version 12.

## Results

### Search Results, Study Selection, Study Characteristics, and Quality Assessment

**Figure 1:**
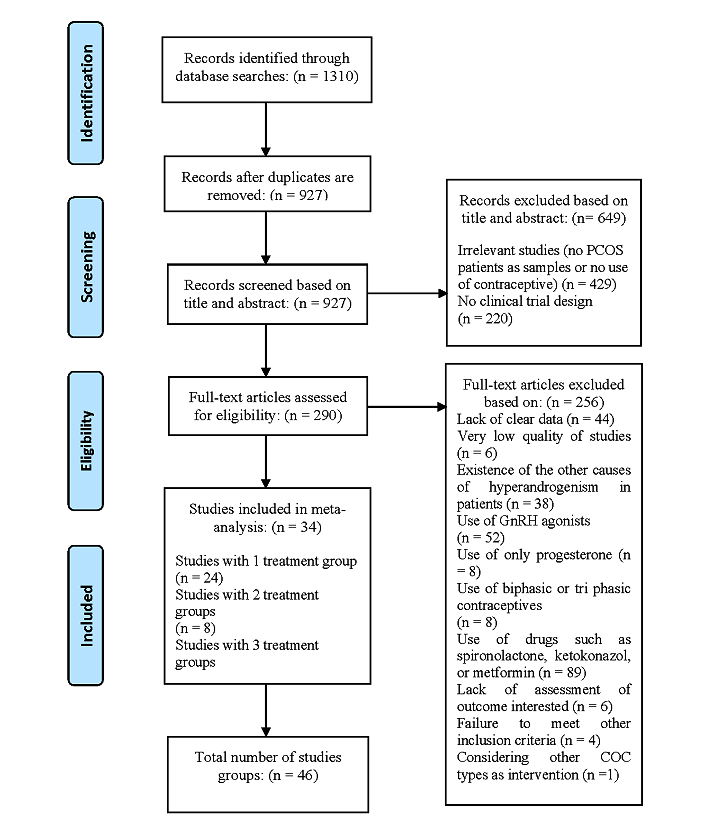
Flow diagram of literature search and study selection. PCOS: polycystic ovary syndrome; GnRH: gonadotropin-releasing hormone; COC: combined oral contraceptives.

A total of 1310 studies were retrieved by searching the electronic databases. After removing duplicates and assessing for quality appraisal and eligibility criteria, 34 studies were selected for the final analyses, which had 46 treatment groups (Figure 1 and Multimedia Appendix 2). Among these, 19 studies were RCTs and 15 studies were NRS. In all, 6 studies were classified as high, 20 as moderate, and 8 as low quality; 6 studies were identified as very low quality and were excluded from the meta-analysis. In most of the included studies (n=25), PCOS was diagnosed by Rotterdam criteria. For other studies, NIH (n=4) and AES criteria (n=2) for diagnosing PCOS were used. Only 2 studies did not report their PCOS criteria. Also, for one study, we used the Homburg criteria for diagnosing PCOS. Ethinyl estradiol (EE) was the estrogenic component of COCs in all studies, whereas the progestin components were CA, DSG, DRSP, or CMA. Of 46 study arms, 20 were exposed to EE 35 µg + CA 2 mg [2,4,10,32-47], 17 to EE 30 µg + DRSP 3 mg [1,4,9,10,19,42,45,48-56], 6 to EE 30 µg + DSG 150 µg and 3 to EE 30 µg + CMA 2 mg (Multimedia Appendix 2) [9,53,57].

The study population consisted of 1224 women with PCOS with a mean age of 24.20 (95% CI 23.19-25.30) years and a mean BMI of 24.42 (95% CI 23.83-25.74) kg/m^2^ (Multimedia Appendix 2). Sufficient data were collected for treatments of 3, 6, and 12 months but not for treatments of 24 months. All hormonal measurements of the studies were performed during the early follicular phase. Only 2 studies did not report days of hormonal assessment [22,58]. The effects of different COC treatments are summarized in Table 1 and Multimedia Appendices 3 and 4.

### Follicle-Stimulating Hormone

A total of 13 studies reported effects of COCs on FSH. No study assessed the effects on FSH of EE + CMA for 3 to 12 months and EE + DSG for 12 months.

The use of EE + CA for 3 months was significantly associated with a decrease in FSH concentrations (WMD=−0.48; 95% CI −0.81 to −0.15), whereas use of EE + DSG or use of EE+ DRSP were not significantly associated. After 6 months of treatment with EE + CA (WMD=−2.33; 95% CI −3.48 to −1.18) and DRSP (WMD=−0.93; 95% CI −1.79 to −0.08), FSH concentrations decreased, but there was no decrease with EE + DSG use. Use of EE + CA for 12 months was associated with a decrease in FSH concentrations (WMD=−4.70; 95% CI −4.98 to −4.42), whereas the use of EE +DRSP was not. A significant heterogeneity was identified among most comparisons made with the FSH concentrations (Table 1 and Multimedia Appendices 3-5).

### Luteinizing Hormone

A total of 18 studies reported the effects of COCs on LH. No study assessed the effects on LH of EE + CMA for 3 to 12 months and EE + DSG for 12 months.

**Figure 2:**
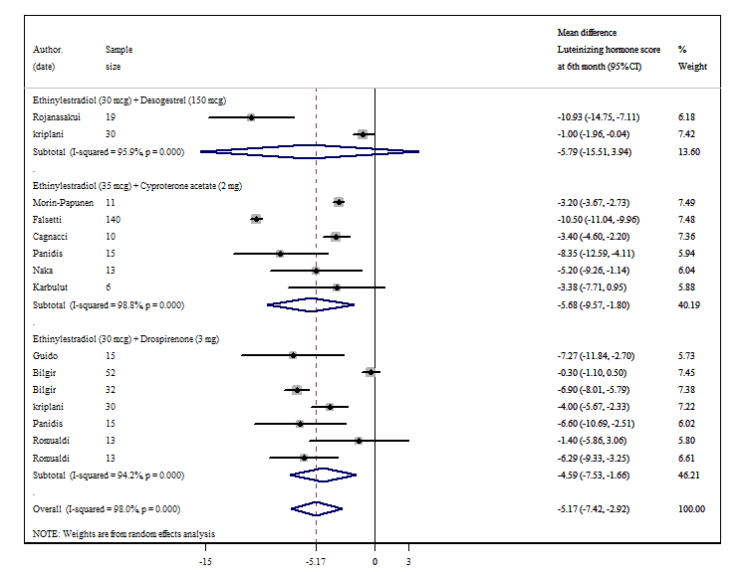
Forest plot of combined oral contraceptives’ effects on luteinizing hormone after 6 months of treatment.

LH concentrations significantly decreased after 3 months of treatment with EE + DSG (WMD=−11.68; 95% CI −13.72 to −9.64) and EE + CA (WMD=−3.57; 95% CI −5.14 to −1.99) but not with EE + DRSP. After 6 months of treatment with EE + CA (WMD=−5.68; 95% CI −9.57 to −1.80) and EE + DRSP (WMD=−4.59; 95% CI −7.53 to −1.66), LH concentrations significantly decreased, whereas no significant decrease in concentration was observed with EE + DSG use (Figure 2). The use of EE + CA (WMD=−11.60; 95% CI −17.60 to −5.60) for 12 months also decreased LH concentrations, whereas use of EE + DRSP did not. There was significant heterogeneity among some comparisons (Table 1 and Multimedia Appendices 3-5).

**Table 1:** Effects of different combined oral contraceptives on hormonal parameters in women with polycystic ovary syndrome. ↑ and ↓ indicate increase and decrease, respectively.

Hormonal parameters		EE^a^ + CA^b^	EE + DRSP^c^	EE + CMA^d^	EE + DSG^e^
**FSH^f^**					
	3 months	↓	NO^g^	N/A^h^	NO
	6 months	↓	↓	N/A	NO
	12 months	↓	NO	N/A	N/A
**LH^i^**					
	3 months	↓	NO	N/A	↓
	6 months	↓	↓	N/A	NO
	12 months	↓	NO	N/A	N/A
**LH-to-FSH ratio**					
	3 months	NO	NO	N/A	NO
	6 months	NO	NO	N/A	NO
	12 months	NO	NO	N/A	N/A
**E2^j^**					
	3 months	↓	NO	N/A	N/A
	6 months	↓	NO	N/A	N/A
	12 months	↓	NO	N/A	N/A
**TT^k^**					
	3 months	↓	↓	↓	↓
	6 months	↓	↓	↓	↓
	12 months	↓	↓	↓	NO
**SHBG^l^**					
	3 months	↑	↑	↑	↑
	6 months	↑	↑	NO	↑
	12 months	↑	↑	↑	↑

^a^EE: ethinyl estradiol.

^b^CA: cyproterone acetate.

^c^DRSP: drospirenone.

^d^CMA: chlormadinone acetate.

^e^DSG: desogestrel.

^f^FSH: follicle-stimulating hormone.

^g^NO: no significant effect.

^h^N/A: not assessed.

^i^LH: luteinizing hormone.

^j^E2: estradiol.

^k^TT: total testosterone.

^l^SHBG: sex hormone–binding globulin.

### Luteinizing Hormone to Follicle-Stimulating Hormone Ratio

A total of 13 studies reported the effects of COCs on LH-to-FSH ratio. No study assessed the effect on LH-to-FSH ratio of EE + CMA for 3 to 12 months and EE + DSG for 12 months.

The use of EE + DSG, EE + CA, and EE + CMA for 3 to 12 months was not associated with any significant change in LH-to-FSH ratio (Table 1 and Multimedia Appendices 3-5).

### Estradiol

A total of 7 studies reported the effects of COCs on E2, whereas no study assessed the effect on E2 of EE + CMA and EE + DSG for 3 to 12 months.

The use of EE + CA for 3 to 12 months significantly decreased the E2 concentrations; WMDs (95% CI) in these durations of follow-ups were −5.62 (−10.49 to −0.75), −28.90 (−31.44 to −26.36), and −32.43 (−46.11 to −18.74), respectively. EE + DRSP use was not associated with any significant change in E2 concentrations. No significant heterogeneity was identified among comparisons (Table 1 and Multimedia Appendices 3-5).

### Total Testosterone

A total of 30 studies reported the effects of COCs on TT.

The use of various COCs, including EE + DSG (WMD=−0.41; 95% CI −0.73 to −0.08), EE + CA (WMD=−0.25; 95% CI −0.29 to −0.21), and EE + DRSP (WMD=−0.22; 95% CI −0.38 to −0.05), was associated with a significant decrease in TT after 3 months of treatment; however, there was no decrease after EE + CMA use. After 6 months of use, all treatments including EE + DSG (WMD=−0.20; 95% CI −0.36 to −0.04), EE + CA (WMD=−0.30; 95% CI −0.44 to −0.16), EE + DRSP (WMD=−0.17; 95% CI −0.23 to −0.11), and EE + CMA (WMD=−0.24; 95% CI −0.37 to −0.11) decreased TT concentrations. The 12-month use of EE + CA (WMD=−0.29; 95% CI −0.54 to −0.04), EE + DRSP (WMD=−0.12; 95% CI −0.22 to −0.03), and EE + CMA (WMD=−0.10; 95% CI −0.17 to −0.03) also decreased TT concentrations, although EE + DSG use was not associated with any significant change in TT. For all comparisons made with TT concentrations, significant heterogeneity was identified (Table 1 and Multimedia Appendices 3-5).

### Sex Hormone–Binding Globulin

A total of 25 studies reported the effects of COCs on SHBG.

Different COCs containing EE + DSG (WMD=99; 95% CI 88.74-109.26), EE + CA (WMD=96.86; 95% CI 47.88-145.84), EE + DRSP (WMD=100.90; 95% CI 12.50-189.30), and EE + CMA (WMD=137.73; 95% CI 89.14-186.32) were associated with increase in SHBG concentrations, following 3 months of treatment. After 6 months of treatment, EE + DSG (WMD=57.35; 95% CI 19.59-95.11), EE + CA (WMD=102.17; 95% CI 82.72-121.63), EE + DRSP (WMD=93.54; 95% CI 63.63-123.45) increased SHBG concentrations, whereas EE + CMA use was not associated with any significant change in SHBG. SHBG concentrations were also increased after 12 months of treatment with all COCs, including EE + DSG (WMD=181.98; 95% CI 20.25-343.71), EE + CA (WMD=162.10; 95% CI 101.63-222.56), EE + DRSP (WMD=89.33; 95% CI 41.45-137.21), and EE + CMA (WMD=9.24; 95% CI 6.65-11.83). There was significant heterogeneity identified among comparisons (Table 1 and Multimedia Appendices 3-5).

### Publication Bias

The results of Egger test showed a significant publication bias for FSH (*P*=.01) and E2 after 6 months (*P*=.07), and corrections were performed on the outcomes. Metatrim showed a change (from mean difference, MD=−1.30; 95% CI −2.14 to −0.46 to MD=−1.33; 95% CI −2.16 to −0. 49) for FSH after 6 months but no change for E2 (MD=−8.96; 95% CI −24.16 to 6.24) in women with PCOS after correcting for publication bias (Multimedia Appendix 6). Other publication biases were not significant.

### Meta-Regression Analysis

We used meta-regression to evaluate heterogeneity induced by variables, including diagnostic criteria of PCOS (Rotterdam, NIH, AES, and other), BMI, and method of assay of different hormones (radioimmunoassay, chemical/electrochemical luminescence, enzyme, and unknown). Our univariate meta-regression analysis showed that BMI has a significant effect on FSH difference at 6th month compared with baseline level (beta=.55; *P*=.096). Diagnostic criteria of PCOS were also a significant source of heterogeneity for FSH difference at the 6th month from the baseline level (*P*=.02) and SHBG difference at the 12th month from the baseline level (beta=−4.21; *P*=.002). The method of assay was also a significant source of heterogeneity for TT difference at the 6th month from the baseline level (beta=−.29; *P*=.007) and SHBG difference at the 6th month from the baseline level (beta=44.45; *P*=.085). None of the potential confounders had any effect on LH and E2 levels. As previously mentioned, for meta-regression, a *P* value <.10 was considered statistically significant.

Our multivariate meta-regression was done only for FSH difference at the 6th month from the baseline level, which had more than one source of heterogeneity: BMI and diagnostic criteria of PCOS. It showed that only diagnostic criteria of PCOS is a significant source of heterogeneity (beta=−4.46; *P*=.059). We did not use multivariate meta-regression for other variables because none of them had more than 1 source for their heterogeneity among the 3 variables, including BMI, diagnostic criteria of PCOS, and method of assay.

### Sensitivity Analysis

The results of metainf showed that there are few studies that can distort the results. Most of the time the point estimates and 95% CIs are in a specified similar limit with others, which showed homogeneity among the studies. We can hence ignore the risk of introducing bias by BMI, diagnostic criteria of PCOS, or method of assay. Details of the sensitivity analysis are presented in Multimedia Appendix 7.

### Risk of Bias Assessment

Multimedia Appendices 8 and 9 show details of risk of bias of published studies. Most RCT studies were at low risk of bias of random sequence generation (52%, 10/19), blinding of participants and personnel (63%, 17/19), and selective outcome reporting (89%, 17/19; in these studies, some biases were more probable such as blinding of outcome assessment and incomplete outcome data (Multimedia Appendix 8). The NRS were not at a high risk of bias. . They had a low risk bias for classification of interventions and selection of reported results (Multimedia Appendix 9).

Generally, most studies had an acceptable validity (low risk of bias), demonstrating high quality of these studies in most aspects. Subgroup analysis based on the risk of bias showed no significant change in outcomes, indicating logical generalizability of these studies.

## Discussion

### Principal Findings

This meta-analysis compared the effects of COCs with progestins containing low androgenic and antiandrogenic activities on the HPG axis in patients with PCOS. A total of 34 studies involving 1224 women was included in this analysis. Findings showed that the use of COCs containing CA was significantly associated with a decrease in gonadotropins (FSH and LH) and E2 concentrations, whereas COCs containing DRSP did not change these parameters. Data were insufficient to assess the effects of COCs containing CMA and DSG on gonadotropins and E2, but in general these products had no significant effects on these hormonal parameters. COCs were not associated with any significant change in the LH-to-FSH ratio. The use of all COCs was associated with an increase in SHBG and decrease in TT levels, except for DSG at 12 month and CMA at 6 month of treatment.

PCOS has a complex pathogenesis and is believed to be a result of disturbances in gonadotropin secretion. Abnormal secretion of gonadotropins, particularly LH, from the pituitary gland leads to abnormal and excessive ovarian theca cell androgens [59].

Estrogen and progestin components of COCs act together to suppress FSH and LH secretion and the midcycle gonadotropin surge by a feedback mechanism, which results in a decrease in ovarian steroidogenesis [49,60,61]. Indeed, suppression of LH is the major mechanism that mediates the effects of these products in PCOS patients [62].

This study showed that COC use was significantly associated with a suppression of gonadotropin (FSH and LH) levels. Duration of treatment is considered to be an important factor in the suppression of gonadotropins. In fact, the use of products containing CA for 3 to 12 months was associated with a decrease in FSH and LH levels, whereas COCs containing DRSP decreased these hormones only after 6 months of treatment. Thus, products containing DRSP generally require a more prolonged usage to suppress the gonadotropins. Data for products containing DSG are limited, but this compound generally had no influence on gonadotropin levels similar to that observed with COCs containing CA and DRSP. COCs containing CA are associated with higher gonadotropin suppression compared with that of other COCs.

No studies assessed the effect of EE + DSG, EE + CMA, and EE + GSD on E2 levels. Therefore, data were available only for evaluating the effect of compounds containing EE 35 µg + CA 2 mg and EE 30 µg + DRSP 3 mg on E2 levels. This analysis demonstrated that COCs containing CA significantly decreased E2 concentrations, whereas COCs containing DRSP exhibited no such effect. Duration of treatment with COCs was not significant on E2 concentrations. This review clearly shows that COCs containing CA are more effective compared with COCs containing DRSP on E2 levels; however, these data are not sufficient to assess the effect of other contraceptives on E2.

Testosterone, a major androgen in women, increases in many PCOS patients. Although all COCs can decrease androgen levels by gonadotropin suppression, contraceptives with antiandrogen progestins have additional specific mechanisms in addition to the main mechanisms to improve HA [16,63]. Therefore, it can be suggested that gonadotropins are independent of sex steroid secretion [64]. Similar to all progestins, antiandrogen progestins inhibit LH and increase clearance of testosterone, which leads to a decrease in androgen levels [65,66]. These newer progestins also exert antiandrogenic effects by competing at the receptor sites with androgens and inhibit 5 alpha-reductase activity [16,65]. Five alpha reductase enzyme catalyzes testosterone to dihydrotestosterone [67]. This key enzyme is necessary for biosynthesis and metabolism of androgens [68]. Adipose tissue is an important source of active steroid production and metabolism. It contains the aromatase enzyme that converts circulating androgens to estrogens. As some estrogens in premenopausal women originate from the peripheral conversion of androgens, the plasma concentrations of estrone and E2 may be significantly correlated with the extent of adipose mass. Obesity is associated with several abnormalities in androgen metabolism [69]. Urinary excretion of SHBG is lower in obese women compared with normal-weight women [70]. Kirschner et al found that menopausal women with abdominal obesity had higher testosterone levels than those with peripheral obesity [71]. Obesity is associated with increased androgen production rate and metabolic clearance rate; however, the main differences are higher estrogen and lower SHBG levels, whereas usually no differences are found in androgen and gonadotropin concentrations [69].

Interestingly, this study showed that all COCs containing CA, DRSP, CMA, and DSG can decrease TT concentrations. The type of COC and duration of treatment with COCs had no significant effects on TT concentrations. A meta-analysis assessed the effect of COCs on testosterone concentrations in healthy women and found that the progestin type of COCs does not affect the testosterone levels [66]; their findings are consistent with those of this meta-analysis. However, they did not evaluate the pituitary hormones and other hormones secreted by the ovary in women with PCOS.

This review also strongly demonstrates that SHBG significantly increased during the use of COCs. Progestin compound and duration of treatment had no important effects on the changes in SHBG levels.

Comparative studies are not adequate to assess the effect of COCs on HPG; therefore, this meta-analysis included NRS and individual arms of RCTs. However, it is well known that meta-analysis of NRS can produce equally or more precise findings for a clinical question compared with meta-analysis of RCTs alone [3,72].

### Limitations

This study has several limitations that should be mentioned. First, there is no single definition for the diagnosis of PCOS and its components. Second, different studies assessed hormonal measurements by different methods. Third, some studies did not assess all hormonal parameters. Finally, some subgroups in this meta-analysis had limited studies to analyze, which can affect the robustness of the results. Therefore, additional studies are required to provide more concrete data to investigate and confirm the accuracy of this conclusion.

There was significant heterogeneity in most outcomes, which can reflect clinical heterogeneity related to variability in PCOS diagnostic criteria; interpretation of laboratory tests; study population, for example, age; BMI; ethnicity or race; and methods used to measure hormones. To deal with significant heterogeneities, we used the random effect model. A majority of studies included in this analysis used the Rotterdam criteria for diagnosing PCOS and a limited number used other diagnostic criteria, such as AES or NIH. Therefore, this meta-analysis has also included PCOS patients with oligomenorrea and cystic ovaries without clinical or biochemical HA. The results of this study showed that diagnostic criteria of PCOS can also be a source of heterogeneity for FSH concentrations, indicating that variability in diagnostic criteria can be a cause of differences in gonadotropin concentrations. However, a sensitivity analysis showed that most of the time point estimates and 95% CIs are in a specified limit similar to those of others; hence, we can ignore the risk of introducing bias by BMI, diagnostic criteria of PCOS, or method of assay. Some included studies reported the early follicular phase as the timing of hormonal measurement without determining its exact time; however, blood samples for all studies were collected at early follicular phase of the spontaneous menstrual cycle or progesterone-induced menstrual bleeding.

To minimize selection bias, study selection was conducted based on the eligibility criteria, which had been accurately determined just before the study. A hand search of the reference lists of all selected papers was also conducted to prevent missing studies.

The pooled estimate of this meta-analysis provides precise results as it has acceptable risk of bias and publication bias; in addition, it included studies conducted among reproductive age women from various regions of the world. Moreover, a subgroup analysis based on the risk of bias was not associated with any significant differences in outcomes. Hence, we can rely on the pooled estimate and the generalizability of these studies.

In this review, the difference between baseline and posttreatment levels was calculated to assess the effects of COCs on the HPG axis. However, subgroup analyses based on different baseline levels and PCOS phenotypes were not performed because of the limitations of the existing studies.

### Conclusions

This meta-analysis indicates that COC use for 3 to 12 months can suppress gonadotropins, leading to a decrease in androgenic profiles in women with PCOS. Although progestin compounds used and duration of treatment were not effective in reducing the circulating levels of androgens and SHBG, they were important in gonadotropin suppression. This study demonstrates that products containing CA have the greatest suppressive effect on gonadotropins and E2, indicating that the use of this compound may be a better alternative for PCOS patients with impaired gonadotropins. However, because of the limitations of the data available for comparison of the effects of all COCs on HPG, the investigators recommend designing further comparative studies.

## Appendix

Multimedia Appendix 1 PRISMA (Preferred Reporting Items checklist for Systematic Reviews and Meta-Analyses) 2009 checklist.

Multimedia Appendix 2 Characteristics of eligible studies included in the meta-analysis.

Multimedia Appendix 3 Effects of EE 35 + CA 2 and EE 30 + DRSP 3 on hormonal parameters.

Multimedia Appendix 4 Effects of EE 30 + CMA 2 and EE 30 + DSG 150 on hormonal parameters.

Multimedia Appendix 5 Forest plots of combined oral contraceptives’ effects on hormonal parameters, including the follicle-stimulating hormone, luteinizing hormone, estradiol, total testosterone, and sex hormone-binding globulin.

Multimedia Appendix 6 Funnel plots of publication bias and related corrections.

Multimedia Appendix 7 The results of sensitivity analysis.

Multimedia Appendix 8 Risk of bias for randomized controlled trials.

Multimedia Appendix 9 Risk of bias for nonrandomized studies.

## Abbreviations

AES: Androgen Excess Society
BMI: body mass index
CA: cyproterone acetate
CMA: chlormadinone acetate
COCs: combined oral contraceptives
CONSORT: Consolidated Standards of Reporting Trials
DRSP: drospirenone
DSG: desogestrel
EE: ethinyl estradiol
E2: estradiol
FSH: follicle-stimulating hormone
GSD: gestodene
HA: hyperandrogenism
HPG: hypothalamic-pituitary-gonadal
LH: luteinizing hormone
LNG: levonorgestrel
MD: mean difference
NIH: National Institutes of Health
NO: no significant effect
NRS: nonrandomized studies
N/A: not assessed
PCOS: polycystic ovary syndrome
PRISMA: Preferred Reporting Items checklist for Systematic Reviews and Meta-Analyses
RCT: randomized controlled trial
SHBG: sex hormone–binding globulin
TT: total testosterone
WMD: weighted mean differences
