# Supplementary material for: Comparing the Effects of Combined Oral Contraceptives Containing Progestins With Low Androgenic and Antiandrogenic Activities on the Hypothalamic-Pituitary-Gonadal Axis in Patients With Polycystic Ovary Syndrome: Systematic Review and Meta-Analysis
Source: JMIR Res Protoc. 2018 Apr 25;7(4):e113. doi: 10.2196/resprot.9024 (PMC5943622; doi:10.2196/resprot.9024)
Supplement: Multimedia Appendix 2 [file resprot_v7i4e113_app2.pdf]

Author. Date	Country	Study design	PCOS diagnostic criteria	N	Age (mean ± SD)	BMI (mean ± SD)	Follow-ups (months)	Intervention	Hormonal measurements	Day of cycle for hormonal measurement ¥	Quality score
**Rojanasakui et al. 1987 [22]**	Thailand	NRS	NA	19	26.58±5.03	25.18±2.62	3, 6	EE 30 + DSG 150	FSH, LH, LH to FSH, TT	N	Moderate
**Nader et al, 1997 [57]**	U.S.A	NRS	NA	16	29.00±1.00	36.80±1.80	6	EE 30 + DSG 150	TT, SHBG	N	Moderate
**Morin et al. 2000 [32]**	Finland	RCT	Homburg	11	29.80±1.00	37.20±1.80	3, 6	EE 35 + CA 2	LH, TT, SHBG	1-7	Moderate
**Armstrong et al. 2001 [33]**	UK	NRS	Rotterdam	11	28.00±2.00	27.30±1.00	3	EE 35 + CA 2	TT, SHBG	Early follicular phase	Moderate
**Falsetti et al. 2001 [34]**	Italy	NRS	Rotterdam	140	24.10±4.90	23.90±3.10	6, 12	EE 35 + CA 2	FSH, LH, LH to FSH, E2, TT, SHBG	5-7	High
**Elter et al. 2002 [2]**	Turkey	RCT	Rotterdam	20	23.45±6.07	21.83±1.40	3	EE 35 + CA 2	LH, TT, SHBG,	Early follicular phase	High
**Mastorakos et al. 2002 [35]**	Greece	RCT	NIH	14	17.53±0.51	25.50±1.79	12	EE 30 + DSG 150	TT, SHBG	3-7	Moderate
**Mastorakos et al. 2002 [35]**	Greece	RCT	NIH	14	17.46±0.43	24.84±1.09	12	EE 35 + CA 2	TT, SHBG	3-7	Moderate
**Cagnacci et al. 2003 [36]**	U.S.A	RCT	Rotterdam	10	21.80±0.80	22.60±0.90	6	EE 35 + CA 2	FSH, LH, LH to FSH, TT, SHBG	4-7	Moderate
**Guido et al. 2004 [48]**	Italy	NRS	Rotterdam	15	25.30±3.51	24.68±5.95	3, 6, 12	EE 30 + DRSP 3	FSH, LH, LH to FSH, TT, SHBG	3-7	Moderate
**Palep-Singh et al. 2005 [19]**	UK	NRS	Rotterdam	17	26.40±5.30	29.00±3.70	6	EE 30 + DRSP 3	TT, SHBG	N	Moderate
**Liqun et al. 2005 [37]**	China	RCT	NIH	25	24.35±5.11	21.81±1.37	6	EE 35 + CA 2	TT, SHBG	3-5	Low
**Pehlivanov et al. 2007 [49]**	Bulgaria	NRS	Rotterdam	20	23.00±4.10	26.45±0.65	6	EE 30 + DRSP 3	TT, SHBG	Early follicular phase	Moderate
**Wu et al. 2008 [38]**	China	RCT	Rotterdam	7	25.00±4.30	25.30±0.80	3	EE 35 + CA 2	LH, TT	3-5	High
**Wu et al. 2008 [38]**	China	RCT	Rotterdam	12	26.10±4.60	21.40±1.60	3	EE 35 + CA 2	LH, TT	3-5	High
**Gregorou et al. 2008 [50]**	Greece	NRS	Rotterdam	52	25.30±6.40	24.70±4.70	3, 6, 12	EE 30 + DRSP 3	LH, TT, SHBG	3-6	Moderate
**Ozdemir et al. 2008 [51]**	Turkey	RCT	Rotterdam	32	22.70±3.80	22.70±3.80	6	EE 30 + DRSP 3	LH, TT, SHBG	2	Moderate
**Gul et al. 2008 [39]**	Turkey	NRS	Rotterdam	30	25.10±6.94	23.85±4.17	3	EE 35 + CA 2	LH, TT,	3-5	Moderate
**Bilgir et al. 2009 [40]**	Turkey	RCT	Rotterdam	20	24.30±5.70	28.20±60	3	EE 35 + CA 2	FSH, LH, LH to FSH, E2	2-5	Low
**Kebapcilar et al. 2009 [41]**	Turkey	RCT	Rotterdam	22	24.10±6.60	27.20±6.20	3	EE 35 + CA 2	FSH, LH, LH to FSH	2-5	Low
**Mahmood et al. 2009 [42]**	Egypt	RCT	AES	21	26.05±2.44	29.42±2.02	3	EE 35 + CA 2	LH	Early follicular phase	Moderate
**Sagsoz et al. 2009 [52]**	Turkey	NRS	Rotterdam	20	25.70±7.20	22.60±4.50	3	EE 30 + DRSP 3	FSH, LH, , LH to FSH, E2, TT, SHBG	2-5	Moderate
**Chen et al. 2010 [43]**	Taiwan	NRS	Rotterdam	56	24.80±4.77	24.33±5.79	3	EE 35 + CA 2	FSH, LH, LH to FSH, E2, TT, SHBG	2-5	High
**Kriplani et al. 2010 [1]**	India	RCT	Rotterdam	30	22.50±4.70	22.70±2.30	6	EE 30 + DRSP 3	FSH, LH, LH to FSH, TT, SHBG	2-5	High
**Kriplani et al. 2010 [1]**	India	RCT	Rotterdam	30	24.00±3.50	24.10±3.40	6	EE 30 + DSG 150	FSH, LH, LH to FSH, TT, SHBG	2-5	High
**De Leo et al. 2010 [58]**	Italy	RCT	Rotterdam	10	23.20±3.40	22.70±2.20	3	EE 30 + DRSP 3	TT, SHBG	6-8	Low
**De Leo et al. 2010 [58]**	Italy	RCT	Rotterdam	10	25.60±4.10	24.10±2.30	3	EE 30 + CMA 2	TT, SHBG	6-8	Low
**De Leo et al. 2010 [58]**	Italy	RCT	Rotterdam	10	23.10±2.60	22.40±3.60	3	EE 30 + DSG 150	TT, SHBG	6-8	Low
**Teede et al. 2010 [44]**	Australia	RCT	NIH	26	33.50±6.70	35.80±1.50	6	EE 35 + CA 2	TT, SHBG	N	Moderate
**Panidis et al. 2011 [45]**	Greece	RCT	NIH	15	20.67±4.13	21.04±1.97	6	EE 35 + CA 2	FSH, LH, LH to FSH, TT,	5-7	Low

									SHBG		
**Panidis et al. 2011 [45]**	Greece	RCT	NIH	15	22.00±2.07	21.69±2.33	6	EE 30 + DRSP 3	FSH, LH, LH to FSH, TT, SHBG	5-7	Low
**Naka et al. 2011 [46]**	Greece	NRS	Rotterdam	13	20.90±3.70	23.00±4.00	6	EE 35 + CA 2	FSH,LH, LH to FSH, TT, SHBG	3-5	Moderate
**Colonna et al. 2011 [53]**	Italy	RCT	Rotterdam	32	25.60±4.40	23.35±3.60	3, 6	EE 30 + DRSP 3	TT, SHBG	3-5	Low
**Colonna et al. 2011 [53]**	Italy	RCT	Rotterdam	27	25.40±3.40	23.27±3.63	3, 6	EE 30 + CMA 2	TT, SHBG	3	Low
**Karabulut et al. 2012 [47]**	Turkey	NRS	Rotterdam	6	24.40±6.30	28.44±4.24	6	EE 35 + CA 2	FSH, LH, LH to FSH, E2, TT	3	Moderate
**Bhattcharia et al. 2012 [10]**	India	RCT	Rotterdam	58	22.24±4.47	25.41±4.49	6, 12	EE 30 + DSG 150	TT, SHBG	2-3	High
**Bhattcharia et al. 2012 [10]**	India	RCT	Rotterdam	56	22.32±4.17	26.41±3.81	6, 12	EE 35 + CA 2	TT, SHBG	2-3	High
**Bhattcharia et al. 2012 [10]**	India	RCT	Rotterdam	57	22.33±4.76	26.47±4.65	6, 12	EE 30 + DRSP 3	TT, SHBG	2-3	High
**Romualdi et al. 2013 [54]**	Italy	RCT	Rotterdam	13	22.92±3.80	22.13±3.34	6, 12	EE 20 + DRSP 3	FSH, LH, , LH to FSH, E2, TT, SHBG	Early follicular phase	Moderate
**Romualdi et al. 2013 [54]**	Italy	RCT	Rotterdam	13	21.92±2.43	22.65±2.75	6, 12	EE 30 + DRSP 3	FSH, LH, , LH to FSH, E2, TT, SHBG	Early follicular phase	Moderate
**Aydin et al. 2013 [55]**	Turkey	NRS	Rotterdam	28	21.40±4.20	21.80±3.40	6	EE 30 + DRSP 3	TT, SHBG	2-5	Moderate
**Kahraman et al. 2014 [4]**	Turkey	RCT	AES	19	22.30±3.20	23.20±3.30	12	EE 35 + CA 2	E2, TT, SHBG	3	Low
**Kahraman et al. 2014 [4]**	Turkey	RCT	AES	20	22.40±2.70	23.20±5.50	12	EE 30 + DRSP 3	E2, TT, SHBG	3	Low
**Macut et al. 2015 [56]**	Serbia	NRS	Rotterdam	12	24.17±4.88	22.05±3.97	12	EE 30 + DRSP 3	TT, SHBG	Early follicular phase	Low
**Yildizhan et al. 2015 [9]**	Turkey	RCT	Rotterdam	60	25.36±2.91	24.82±1.08	6, 12	EE 30 + DRSP 3	TT, SHBG	Early follicular phase	Moderate
**Yildizhan et al. 2015 [9]**	Turkey	RCT	Rotterdam	60	24.82±3.20	23.56±3.32	6, 12	EE 30 + CMA 2	TT, SHBG	Early follicular phase	Moderate

Abbreviations: N, not motioned; n, sample size; SD, standard deviation;BMI, Body mass Index; CMA, Chlormadinone acetate; EE, Ethinyl Estradiol; DRSP, drospirenone; DSG, desogestrel; CA, cyproterone acetate; RCT, Randomized controlled trial; NRS, non-randomized studies

¥: Blood samples were collected at early follicular phase of the spontaneous menstrual cycle or progesterone-induced menstrual bleeding

References:

22. Rojanasakul A, Sirimongkolkasem R, Piromsawasdi S, Sumavong V, Chailurkit L-o, Chaturachinda K. Effects of combined ethinylestradiol, and desogestrel on hormone profiles and sex hormone binding globulin in women with polycystic ovarian disease. Contraception 1987 Dec; 36(6):633-640. PMID: 2965636

57. Nader S, Riad-Gabriel MG, Saad MF. The Effect of a Desogestrel-Containing Oral Contraceptive on Glucose Tolerance and Leptin Concentrations in Hyperandrogenic Women. The Journal of Clinical Endocrinology & Metabolism 1997 Sep; 82(9):3074-3077. PMID: 9284746

32. Morin-Papunen LC, Vauhkonen I, Koivunen RM, Ruokonen A, Martikainen HK, Tapanainen JS. Endocrine and Metabolic Effects of Metformin Versus Ethinyl Estradiol-Cyproterone Acetate in Obese Women with Polycystic Ovary Syndrome: A Randomized Study 1. The Journal of Clinical Endocrinology & Metabolism 2000 Sep; 85(9):3161-3168. PMID: 10999803

33. Armstrong VL, Wiggam MI, Ennis CN, Sheridan B, Traub AI, Atkinson AB, Bell PM: Insulin action and insulin secretion in polycystic ovary syndrome treated with ethinyl oestradiol/cyproterone acetate. QJM 2001, 94(1):31-37.

34. Falsetti L, Gambera A, Tisi G. Efficacy of the combination ethinyl oestradiol and cyproterone acetate on endocrine, clinical and ultrasonographic profile in polycystic ovarian syndrome. Human Reproduction 2001, 16(1):36-42. PMID: 11139533

2. Elter K, Imir G, Durmusoglu F. Clinical, endocrine and metabolic effects of metformin added to ethinyl estradiol-cyproterone acetate in non-obese women with polycystic ovarian syndrome: a randomized controlled study. Human Reproduction 2002 Jul; 17(7):1729-1737. PMID: 12093831

35. Mastorakos G, Koliopoulos C, Creatsas G. Androgen and lipid profiles in adolescents with polycystic ovary syndrome who were treated with two forms of combined oral contraceptives. Fertility and sterility 2002 May; 77(5):919-927. PMID: 12009344

36. Cagnacci A, Paoletti AM, Renzi A, Orru M, Pilloni M, Melis GB, Volpe A. Glucose metabolism and insulin resistance in women with polycystic ovary syndrome during therapy with oral contraceptives containing cyproterone acetate or desogestrel. The Journal of Clinical Endocrinology & Metabolism 2003 Aug; 88(8):3621-3625. PMID: 12915645

48. Guido M, Romualdi D, Giuliani M, Suriano R, Selvaggi L, Apa R, Lanzone A. Drospirenone for the treatment of hirsute women with polycystic ovary syndrome: a clinical, endocrinological, metabolic pilot study. The Journal of Clinical Endocrinology & Metabolism 2004 Jun; 89(6):2817-2823. PMID: 15181063

19. Palep-Singh M, Mook K, Barth J, Balen A. An observational study of YasminÂ® in the management of women with polycystic ovary syndrome. Journal of Family Planning and Reproductive Health Care 2004 Jul; 30(3):163-165. PMID: 15222920

37. Liqun L, Yi L, Yongyu S, Kan T. Effects of metformin combined with cyproterone acetate on clinical features, endocrine and metabolism of non-obese women with polycystic ovarian syndrome. Journal of Huazhong University of Science and Technology [Medical Sciences] 2005 Apr; 25(2):194-197. Doi: https://doi.org/10.1007/BF02873575

49. Pehlivanov B, Mitkov M. Efficacy of an oral contraceptive containing drospirenone in the treatment of women with polycystic ovary syndrome. The European Journal of Contraception & Reproductive Health Care 2007 Mar; 12(1):30-35. PMID: 17455042

38. Wu J, Zhu Y, Jiang Y, Cao Y. Effects of metformin and ethinyl estradiol-cyproterone acetate on clinical, endocrine and metabolic factors in women with polycystic ovary syndrome. Gynecological Endocrinology 2008 Jul; 24(7):392-398. PMID: 18608522
50. Gregoriou O, Papadias K, Konidaris S, Bakalianou K, Salakos N, Vrachnis N, Creatsas G. Treatment of hirsutism with combined pill containing drospirenone. Gynecological Endocrinology 2008 Apr; 24(4):220-223. PMID: 18382909
51. Ozdemir S, Görkemli H, Gezginç K, Ozdemir M, Kiyici A. Clinical and metabolic effects of medroxyprogesterone acetate and ethinyl estradiol plus drospirenone in women with polycystic ovary syndrome. International Journal of Gynecology & Obstetrics 2008 Oct; 103(1):44-49. PMID: 18635183
39. Gul OB, Somunkiran A, Yucel O, Demirci F, Ozdemir I. The effect of ethinyl estradiol-cyproterone acetate treatment on homocysteine levels in women with polycystic ovary syndrome. Archives of gynecology and obstetrics 2008 Jan; 277(1):25-30. PMID: 17618446
40. Bilgir O, Kebapcilar L, Taner C, Bilgir F, Kebapcilar A, Bozkaya G, Yildiz Y, Yuksel A, Sari I. The effect of ethinylestradiol (EE)/cyproterone acetate (CA) and EE/CA plus metformin treatment on adhesion molecules in cases with polycystic ovary syndrome (PCOS). Internal Medicine 2009 Jul; 48(14):1193-1199. PMID: 19602786
41. Kebapcilar L, Yuksel A, Bozkaya G, Taner CE, Kebapcilar AG, Bilgir O, Alacacioglu A, Sari I. Effects of an EE/CA compared with EE/CA-metformin on serum ADMA levels in women with polycystic ovary syndrome. Central European journal of medicine 2009 Dec; 4(4):423-427. Doi: https://doi.org/10.2478/s11536-009-0074-x
42. Mahmood M, El-Kattan EA, El-Aal HA, El Lithy A, Ghamry NK, Sheta M. Evaluation of the Clinical and Biochemical Effects of Medical Therapy in Women with Polycystic Ovary Syndrome. 2009 Sep; 77(2): 59-67
52. Sağsöz N, Orbak Z, Noyan V, Yücel A, Uçar B, Yildiz L. The effects of oral contraceptives including low-dose estrogen and drospirenone on the concentration of leptin and ghrelin in polycystic ovary syndrome. Fertility and sterility 2009 Aug; 92(2):660-666. PMID: 18973889
43. Chen M-J, Yang W-S, Chen H-F, Kuo J-J, Ho H-N, Yang Y-S, Chen S-U. Increased follistatin levels after oral contraceptive treatment in obese and non-obese women with polycystic ovary syndrome. Human Reproduction 2010 Mar; 25(3):779-785. PMID: 20093255
1. Kriplani A, Periyasamy AJ, Agarwal N, Kulshrestha V, Kumar A, Ammini AC. Effect of oral contraceptive containing ethinyl estradiol combined with drospirenone vs. desogestrel on clinical and biochemical parameters in patients with polycystic ovary syndrome. Contraception 2010 Aug; 82(2):139-146. PMID: 20654754
58. De Leo V, Di Sabatino A, Musacchio MC, Morgante G, Scolaro V, Cianci A, Petraglia F. Effect of oral contraceptives on markers of hyperandrogenism and SHBG in women with polycystic ovary syndrome. Contraception 2010 Sep; 82(3):276-280. PMID: 20705157
44. Teede HJ, Meyer C, Hutchison SK, Zoungas S, McGrath BP, Moran LJ. Endothelial function and insulin resistance in polycystic ovary syndrome: the effects of medical therapy. Fertility and sterility 2010 Jan; 93(1):184-191. PMID: 19019358
45. Panidis D, Georgopoulos NA, Piouka A, Katsikis I, Saltamavros AD, Decavalas G, Diamanti-Kandarakis E. The impact of oral contraceptives and metformin on anti-Mullerian hormone serum levels in women with polycystic ovary syndrome and biochemical hyperandrogenemia. Gynecological Endocrinology 2011 Aug; 27(8):587-592. PMID: 20836726
46. Naka KK, Kalantaridou SN, Bechlioulis A, Kravariti M, Kazakos N, Katsouras CS, Tsatsoulis A, Michalis LK. Effect of ethinylestradiol/cyproterone acetate on endothelial function

in young non-obese women with polycystic ovary syndrome: a pilot study. Gynecological Endocrinology 2011 Sep; 27(9):615-621. PMID: 21329419
53. Colonna L, Pacifico V, Lello S, Sorge R, Raskovic D, Primavera G. Skin improvement with two different oestroprogestins in patients affected by acne and polycystic ovary syndrome: clinical and instrumental evaluation. Journal of the European Academy of Dermatology and Venereology 2012 Nov; 26(11):1364-1371. PMID: 22011217
47. Karabulut A, Demirlenk S, Sevket O. Effects of ethinyl estradiol-cyproterone acetate treatment on metabolic syndrome, fat distribution and carotid intima media thickness in polycystic ovary syndrome. Gynecological Endocrinology 2012 Apr; 28(4):245-8. PMID: 21961995
10. Bhattacharya SM, Jha A. Comparative study of the therapeutic effects of oral contraceptive pills containing desogestrel, cyproterone acetate, and drospirenone in patients with polycystic ovary syndrome. Fertility and sterility 2012 Oct; 98(4):1053-1059. PMID: 22795636
54. Romualdi D, De Cicco S, Busacca M, Gagliano D, Lanzone A, Guido M. Clinical efficacy and metabolic impact of two different dosages of ethinyl-estradiol in association with drospirenone in normal-weight women with polycystic ovary syndrome: a randomized study. Journal of endocrinological investigation 2013 Sep; 36(8):636-641. PMID: 24105072
55. Aydin K, Cinar N, Aksoy DY, Bozdag G, Yildiz BO. Body composition in lean women with polycystic ovary syndrome: effect of ethinyl estradiol and drospirenone combination. Contraception 2013 Mar; 87(3):358-362. PMID: 22898361
4. Kahraman K, Sükür YE, Atabekoğlu CS, Ateş C, Taşkın S, Cetinkaya SE, Tolunay HE, Ozmen B, Sönmezer M, Berker B. Comparison of two oral contraceptive forms containing cyproterone acetate and drospirenone in the treatment of patients with polycystic ovary syndrome: a randomized clinical trial. Archives of gynecology and obstetrics 2014 Aug; 290(2):321-328. PMID: 24676694
