# Supplementary material for: Comparing the Effects of Combined Oral Contraceptives Containing Progestins With Low Androgenic and Antiandrogenic Activities on the Hypothalamic-Pituitary-Gonadal Axis in Patients With Polycystic Ovary Syndrome: Systematic Review and Meta-Analysis
Source: JMIR Res Protoc. 2018 Apr 25;7(4):e113. doi: 10.2196/resprot.9024 (PMC5943622; doi:10.2196/resprot.9024)
Supplement: Multimedia Appendix 3 [file resprot_v7i4e113_app3.pdf]

Type of analysis	Number of treatment groups	Number of subjects	MD (95% CI)	Weight (%)	Heterogeneity (I2) (%)
**EE (35 μg) / CA (2 mg)**					
**FSH Subgroups:**					
FSH after 3 months of treatment	2	86	-0.48 (-0.81, -0.15) †	61.39	0
FSH after 6 months of treatment	5	184	-2.33 (-3.48, -1.18) †	33.03	93.2 #
FSH after 12 months of treatment	1	140	-4.70 (-4.98, -4.42) †	73.31	0
**LH Subgroups:**					
LH after 3 months of treatment	7	157	-3.57 (-5.14, -1.99) †	58.67	90.01 #
LH after 6 months of treatment	6	195	-5.68 (-9.57, -1.80) †	67.88	67.88 #
LH after 12 months of treatment	1	140	-11.60 (-17.60, -5.60) †	35.21	
LH to FSH ratio subgroups:					
LH to FSH ratio after 3 months of treatment	2	86	-0.80 (-3.41, 1.81)	39.95	0
LH to FSH ratio after 6 months of treatment	5	184	-0.73 (-2.72, 1.26)	33.96	0
LH to FSH ratio after 12 months of treatment	1	140	-1.36 (-4.20, 1.48) †	35.78	0
**E2 Subgroups:**					
E2 after 3 months of treatment	1	56	-5.62 (-10.49, -0.75) †	69.51	0
E2 after 6 months of treatment	2	146	-28.90 (-31.44, -26.36) †	75.84	0
E2 after 12 months of treatment	2	159	-32.43 (-46.11, -18.74) †	33.51	69.4 #
**TT Subgroups:**					
TT after 3 months of treatment	7	147	-0.25 (-0.29, -0.21) †	27.77	42.8% #
TT after 6 months of treatment	9	302	-0.30 (-0.44, -0.16) †	45.36	96.2% #
TT after 12 months of treatment	4	229	-0.29 (-0.54, -0.04) †	38.14	97.9% #
**SHBG Subgroups:**					
SHBG after 3 months of treatment	4	98	96.86 (47.87, 145.84)†	27.08	98.6% #
SHBG after 6 months of treatment	8	296	102.17 (82.72, 121.63)†	49.60	98.4 #
SHBG after 12 months of treatment	4	229	162.10 (101.63, 222.56)†	33.09	99.7 #
**EE (30 μg) + DRSP (3 mg)**					
**FSH Subgroups:**					
FSH after 3 months of treatment	3	87	-0.001 (-0.51, 0.50)	25.93	74.2 #
FSH after 6 months of treatment	7	170	-0.93 (-1.79, -0.08) †	51.92	80 #
FSH after 12 months of treatment	4	93	-0.41 (-0.88, 0.06)	26.69	0
**LH Subgroups:**					
LH after 3 months of treatment	3	87	-2 (-4.62, 0.62)	8.88	79.2 #
LH after 6 months of treatment	7	170	-4.59 (-7.53, -1.66) †	23.15	94.2 #
LH after 12 months of treatment	4	93	-1.43 (-5.85, 2.99)	64.79	0
LH to FSH ratio subgroups:					
LH to FSH ratio after 3 months of treatment	3	87	-0.40 (-2.71, 1.90)	51.14	0
LH to FSH ratio after 6 months of treatment	7	170	-0.65 (-2.25, 0.95)	52.53	0
LH to FSH ratio after 12 months of treatment	4	93	-0.52 (-2.64, 1.60)	64.22	0
**E2 Subgroups:**					
E2 after 3 months of treatment	2	72	0.12 (-7.23, 7.48)	30.49	0
E2 after 3 months of treatment	4	110	-2.00 (-6.50, 2.50)	24.16	0
E2 after 3 months of treatment	4	98	3.91 (-26.03, 33.85)	66.49	98.5 #
**TT subgroups:**					
TT after 3 months of treatment	5	129	-0.22 (-0.38, -0.05) †	44.74	96.1 #
TT after 6 months of treatment	12	324	-0.17 (-0.23, -0.11) †	34.80	85.6 #
TT after 12 months of treatment	8	242	-0.12 (-0.22, -0.03) †	27.94	80.9 #
**SHBG Subgroups:**					
SHBG after 3 months of treatment	5	129	100.90 (12.50, 189.30) †	44.28	99.7 #
SHBG after 6 months of treatment	13	384	93.54 (63.63, 123.45) †	31.92	99.5 #
SHBG after 12 months of treatment	8	242	89.33 (41.45, 137.21) †	28.21	99.4 #

Abbreviations: EE, Ethinyl estradiol; CA, cyproterone acetate; DRSP,drospirenone; MD, mean difference; (95% CI), 95% confidence interval; FSH, Follicle-stimulating hormone; LH, Luteinizing hormone; E2, estradiol; TT, total testosterone; SHBG, sex hormone binding globulin.

† Significant values of MD and 95% CI

Significant heterogeneity at the 0.1 level (alpha).
