# Supplementary material for: Comparing the Effects of Combined Oral Contraceptives Containing Progestins With Low Androgenic and Antiandrogenic Activities on the Hypothalamic-Pituitary-Gonadal Axis in Patients With Polycystic Ovary Syndrome: Systematic Review and Meta-Analysis
Source: JMIR Res Protoc. 2018 Apr 25;7(4):e113. doi: 10.2196/resprot.9024 (PMC5943622; doi:10.2196/resprot.9024)
Supplement: Multimedia Appendix 4 [file resprot_v7i4e113_app4.pdf]

Type of analysis	Number of treatment groups	Number of subjects	MD (95% CI)	Weight (%)	Heterogeneity (I2) (%)
**EE (30 µg) +CMA (2 mg)**					
**FSH subgroups:**					
FSH after 3 months of treatment	-----	-----	-----	-----	-----
FSH after 6 months of treatment	-----	-----	-----	-----	-----
FSH after 12 months of treatment	-----	-----	-----	-----	-----
**LH subgroups:**					
LH after 3 months of treatment	-----	-----	-----	-----	-----
LH after 6 months of treatment	-----	-----	-----	-----	-----
LH after 12 months of treatment	-----	-----	-----	-----	-----
LH to FSH ratio subgroups:					
LH to FSH ratio after 3 months of treatment	-----	-----	-----	-----	-----
LH to FSH ratio after 6 months of treatment	-----	-----	-----	-----	-----
LH to FSH ratio after 12 months of treatment	-----	-----	-----	-----	-----
**E2 Subgroups:**					
E2 after 3 months of treatment	-----	-----	-----	-----	-----
E2 after 6 months of treatment	-----	-----	-----	-----	-----
E2 after 12 months of treatment	-----	-----	-----	-----	-----
**TT Subgroups:**					
TT after 3 months of treatment	2	37	-0.26 (-0.53, 0.02) †	24.63	97.20 #
TT after 6 months of treatment	1	27	-0.24 (-0.37, -0.11) †	1.01	0
TT after 12 months of treatment	1	60	-0.10 (-0.17, -0.03) †	9.15	0
**SHBG Subgroups:**					
SHBG after 3 months of treatment	2	37	137.73 (89.14, 186.32) †	21.73	98.5 #
SHBG after 6 months of treatment	2	87	74.55 (-62.93, 212.03)	14.94	99.9 #
SHBG after 12 months of treatment	1	60	9.24 (6.65, 11.83) †	31.37	0
**EE (30 µg) + DSG (150 µg)**					
**FSH subgroups:**					
FSH after 3 months of treatment	1	19	0.37 (-0.35, 1.09)	12.67	0
FSH after 6 months of treatment	2	49	-0.22 (-0.98, 0.55)	15.05	3.2
FSH after 12 months of treatment	-----	-----	-----	-----	-----
**LH subgroups:**					
LH after 3 months of treatment	1	19	-11.68 (-13.72, -9.64) †	1.12	0
LH after 6 months of treatment	2	49	-5.79 (-15.51, 3.94)	8.97	95.9 #
LH after 12 months of treatment	-----	-----	-----	-----	-----
LH to FSH ratio subgroups:					
LH to FSH ratio after 3 months of treatment	2	19	-3.11 (-8.63, 2.41)	8.91	0
LH to FSH ratio after 6 months of treatment	2	49	-0.95 (-4.11, 2.20)	13.50	0
LH to FSH ratio after 12 months of treatment	1	30	-1.13 (-3.86, 1.59)	6.85	
**E2 subgroups:**					
E2 after 3 months of treatment	-----	-----	-----	-----	-----
E2 after 6 months of treatment	-----	-----	-----	-----	-----
E2 after 12 months of treatment	-----	-----	-----	-----	-----
**TT subgroups:**					
TT after 3 months of treatment	2	29	-0.41 (-0.73, -0.08) †	2.87	85.80 #
TT after 6 months of treatment	4	123	-0.20 (-0.36, -0.04) †	18.84	93.60 #
TT after 12 months of treatment	2	72	-0.23 (-0.47, 0.02)	24.78	95.90 #
**SHBG Subgroups:**					
SHBG after 3 months of treatment	1	10	99 (88.74, 109.26) †	6.92	0
SHBG after 6 months of treatment	2	88	57.35 (19.59, 95.11) †	3.55	95.70 #
SHBG after 12 months of treatment	2	72	181.98 (20.25, 343.71) †	7.32	99.80 #

Abbreviations: EE, Ethinyl estradiol; CMA, Chlormadinone acetate; DSG, desogestrel; MD, mean difference; (95% CI), 95% confidence interval; FSH, Follicle-stimulating hormone; LH, Luteinizing hormone; E2, estradiol; TT, total testosterone; SHBG, sex hormone binding globulin

-----, no data

† Significant values of MD and 95% CI

#Significant heterogeneity at the 0.1 level (alpha).
