# Supplementary material for: Comparing the Effects of Combined Oral Contraceptives Containing Progestins With Low Androgenic and Antiandrogenic Activities on the Hypothalamic-Pituitary-Gonadal Axis in Patients With Polycystic Ovary Syndrome: Systematic Review and Meta-Analysis
Source: JMIR Res Protoc. 2018 Apr 25;7(4):e113. doi: 10.2196/resprot.9024 (PMC5943622; doi:10.2196/resprot.9024)

Multimedia Appendix 5: (Forest plots of COCs effects on hormonal parameters including FSH, LH to FSH ratio, E2, TT and SHBG)

Figure 1. Forest plot of COCs effects on FSH after 3 months of treatment.

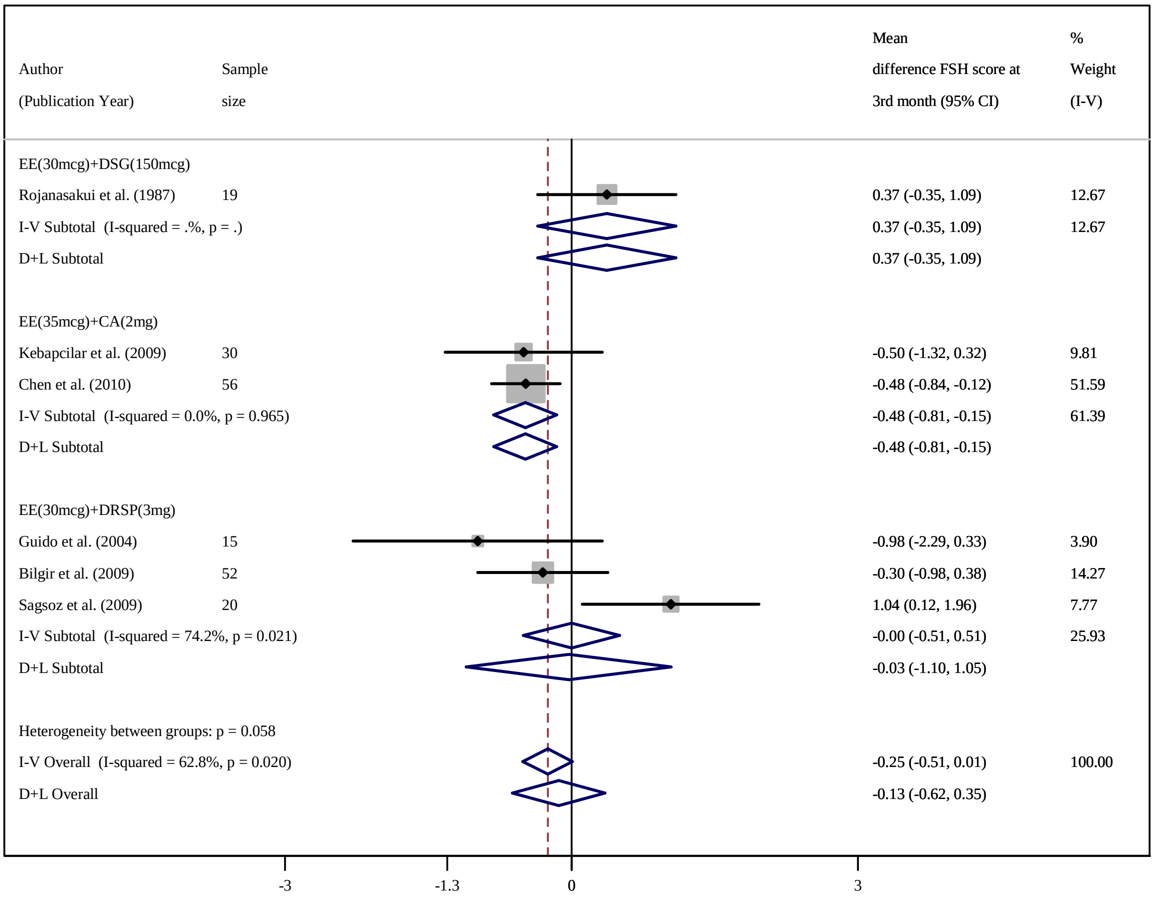

Figure 2. Forest plot of COCs effects on FSH after 6 months of treatment.

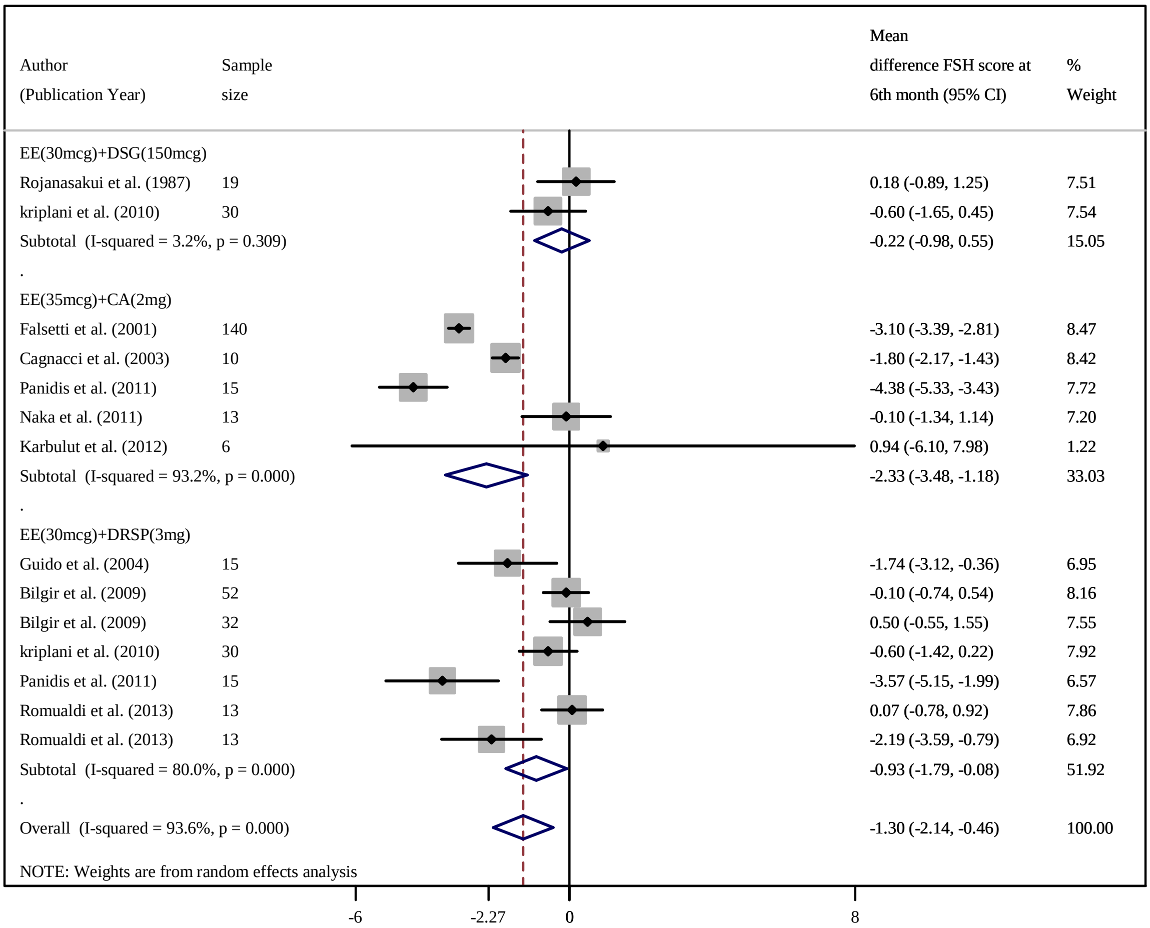

Figure 3. Forest plot of COCs effects on FSH after 12 months of treatment.

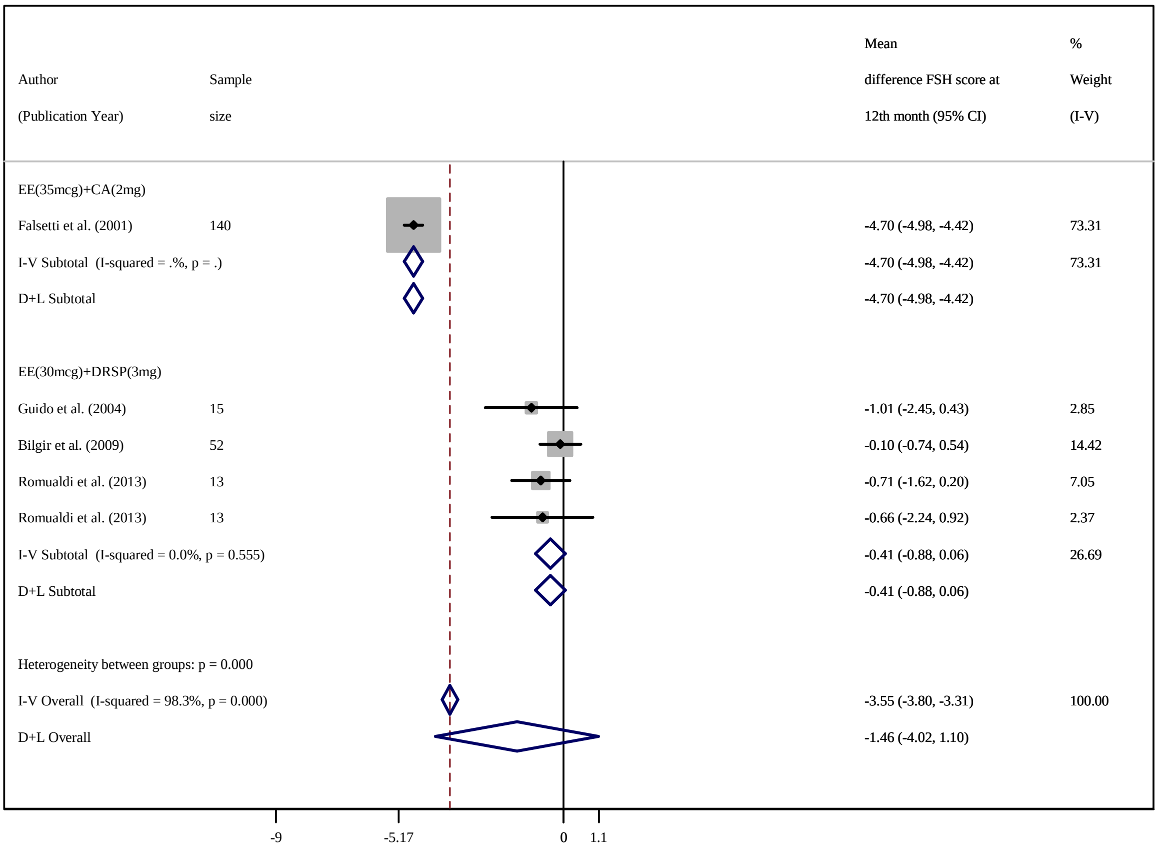

Figure 4. Forest plot of COCs effects on LH after 3 months of treatment.

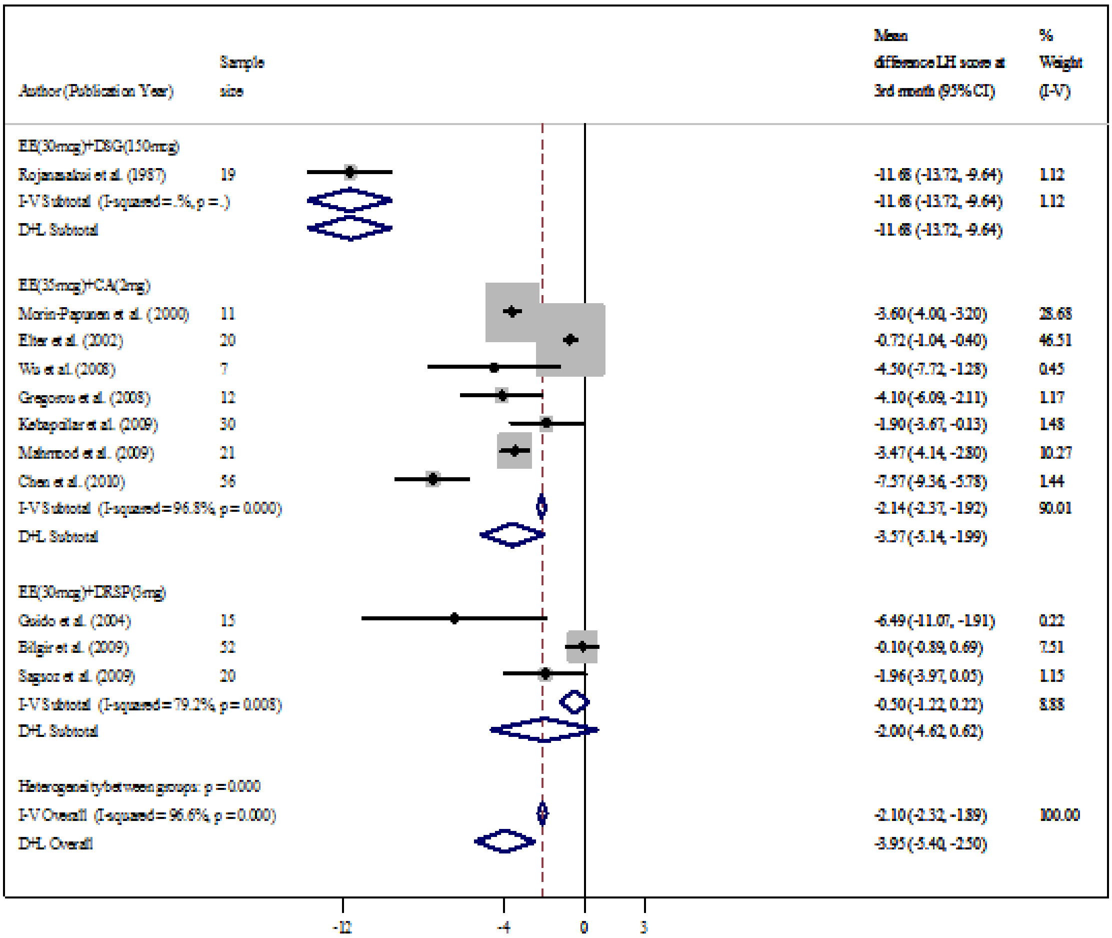

Figure 5. Forest plot of COCs effects on LH after 12 months of treatment.

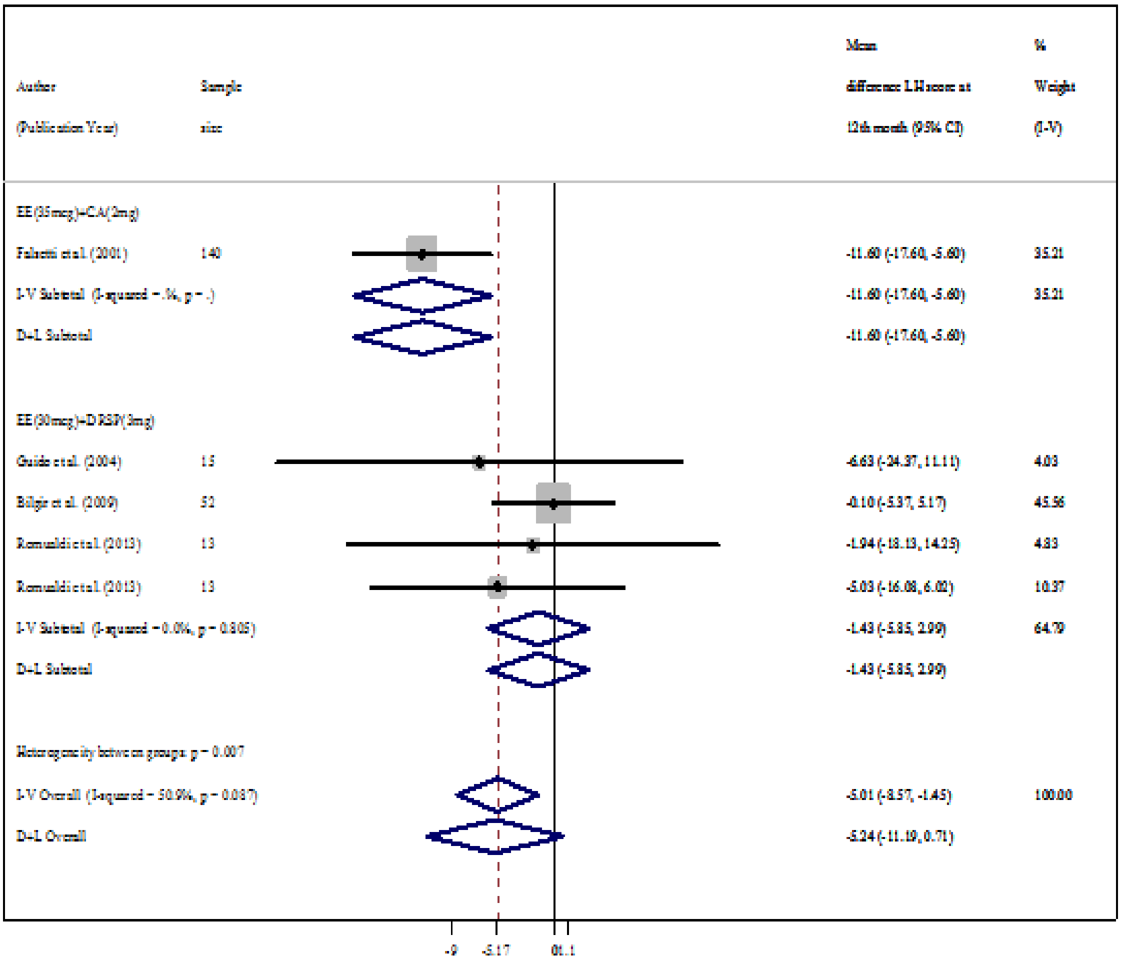

Figure 6. Forest plot of COCs effects on LH to FSH ratio after 3 months of treatment.

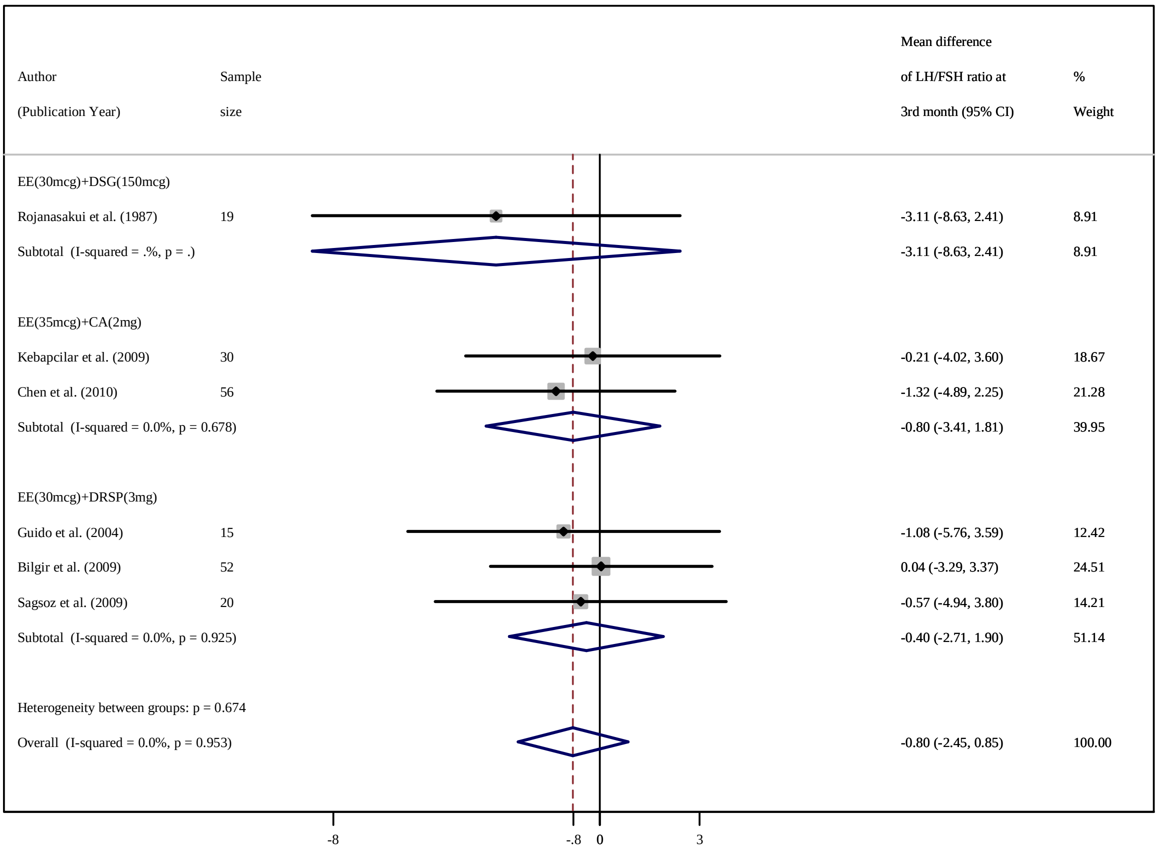

Figure 7. Forest plot of COCs effects on LH to FSH ratio after 6 months of treatment.

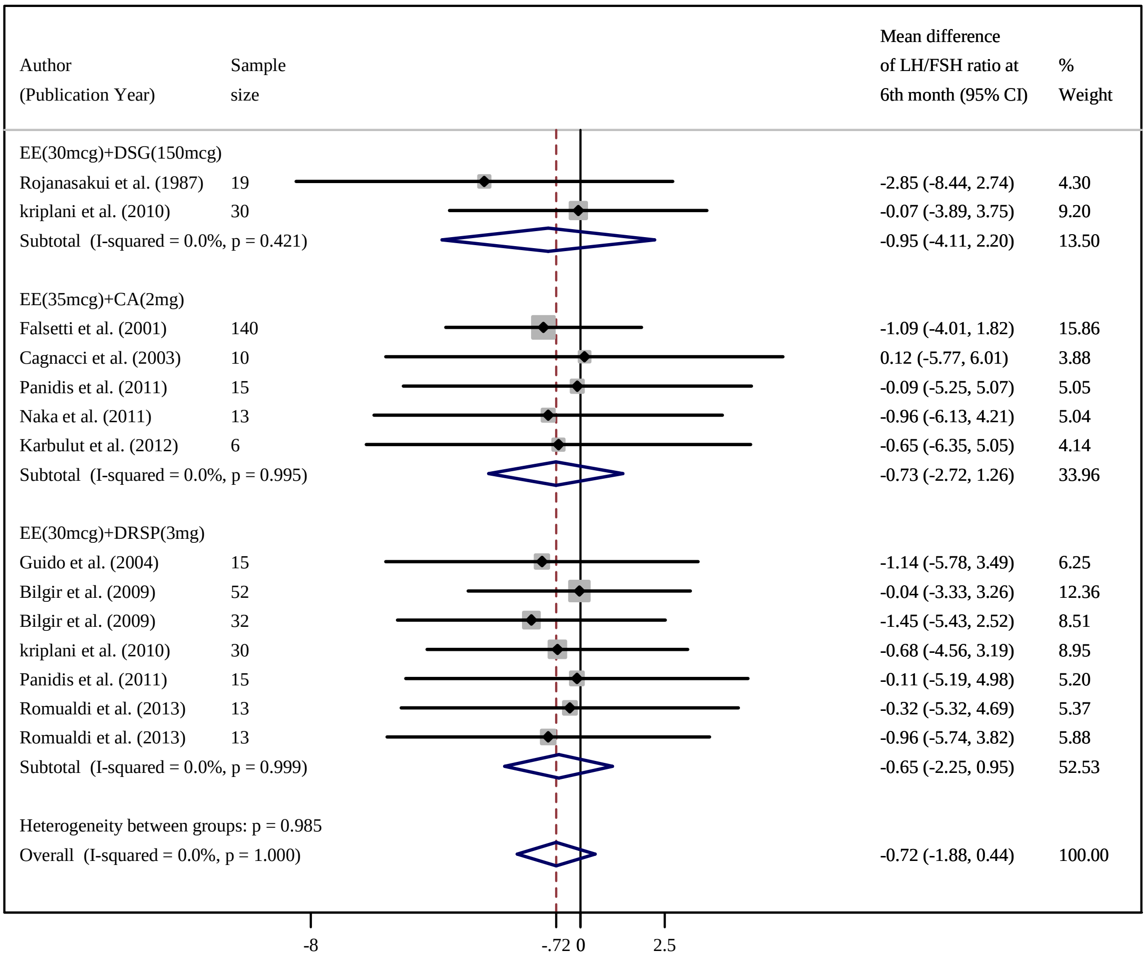

Figure 8. Forest plot of COCs effects on LH to FSH ratio after 12 months of treatment.

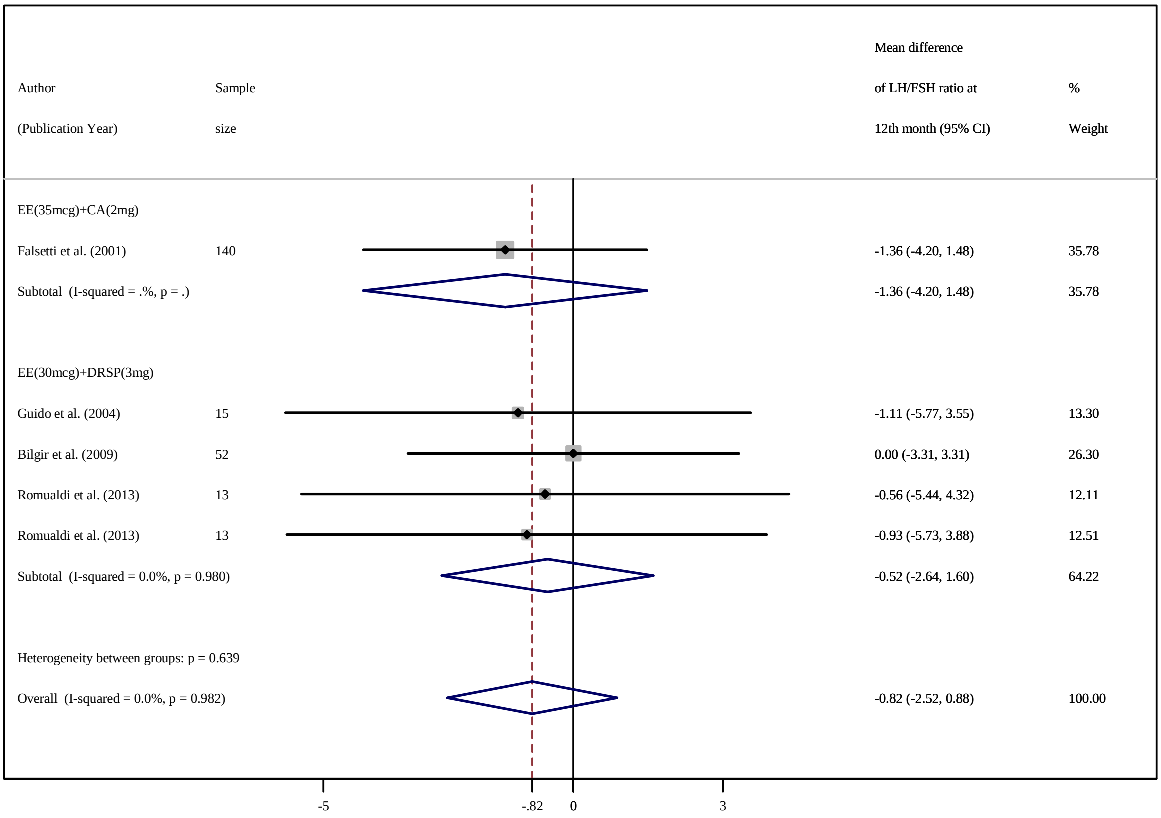

Figure 9. Forest plot of COCs effects on E2 after 3 months of treatment.

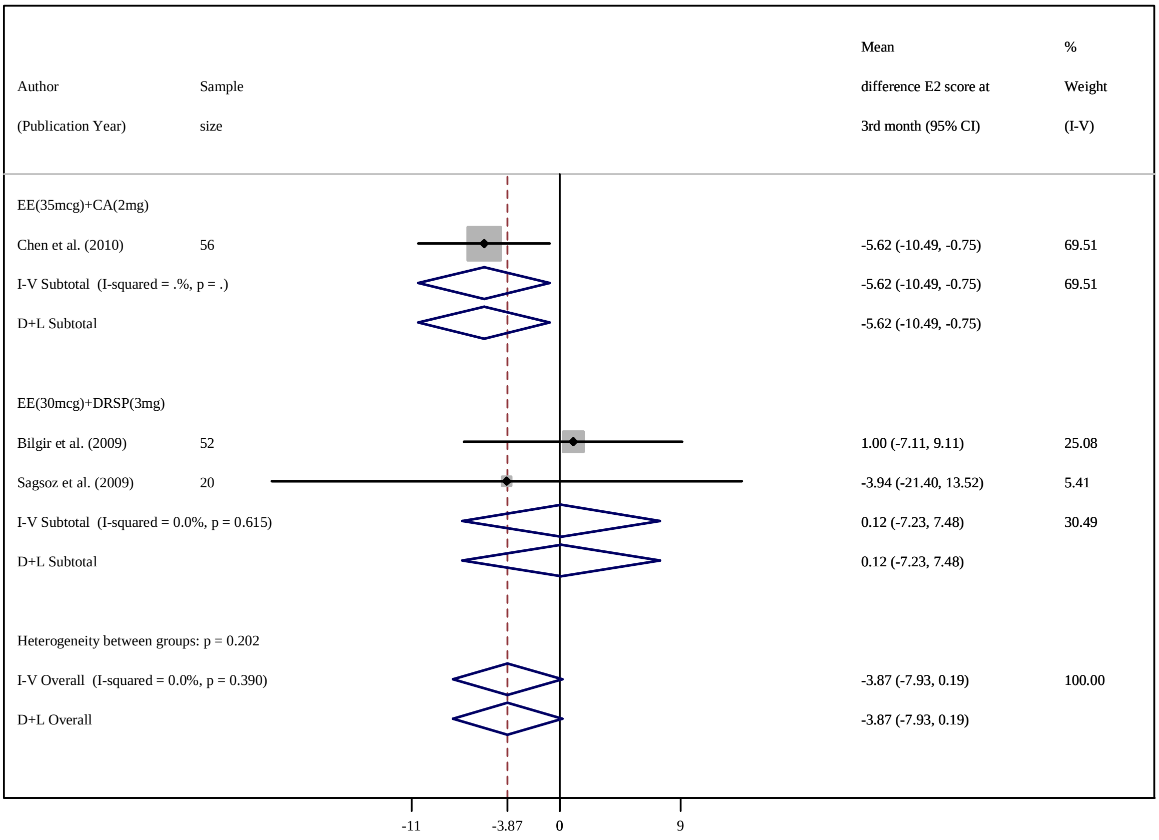

Figure 10. Forest plot of COCs effects on E2 after 6 months of treatment.

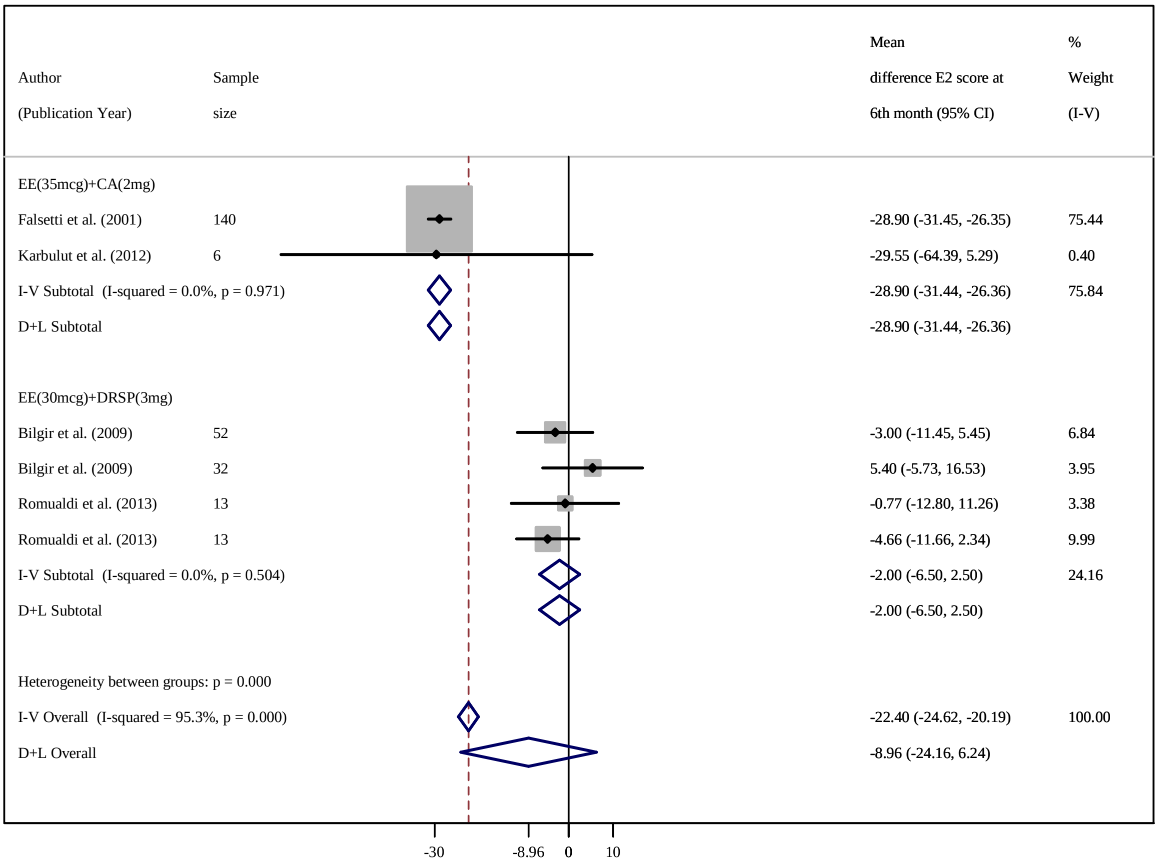

Figure 11. Forest plot of COCs effects on E2 after 12 months of treatment.

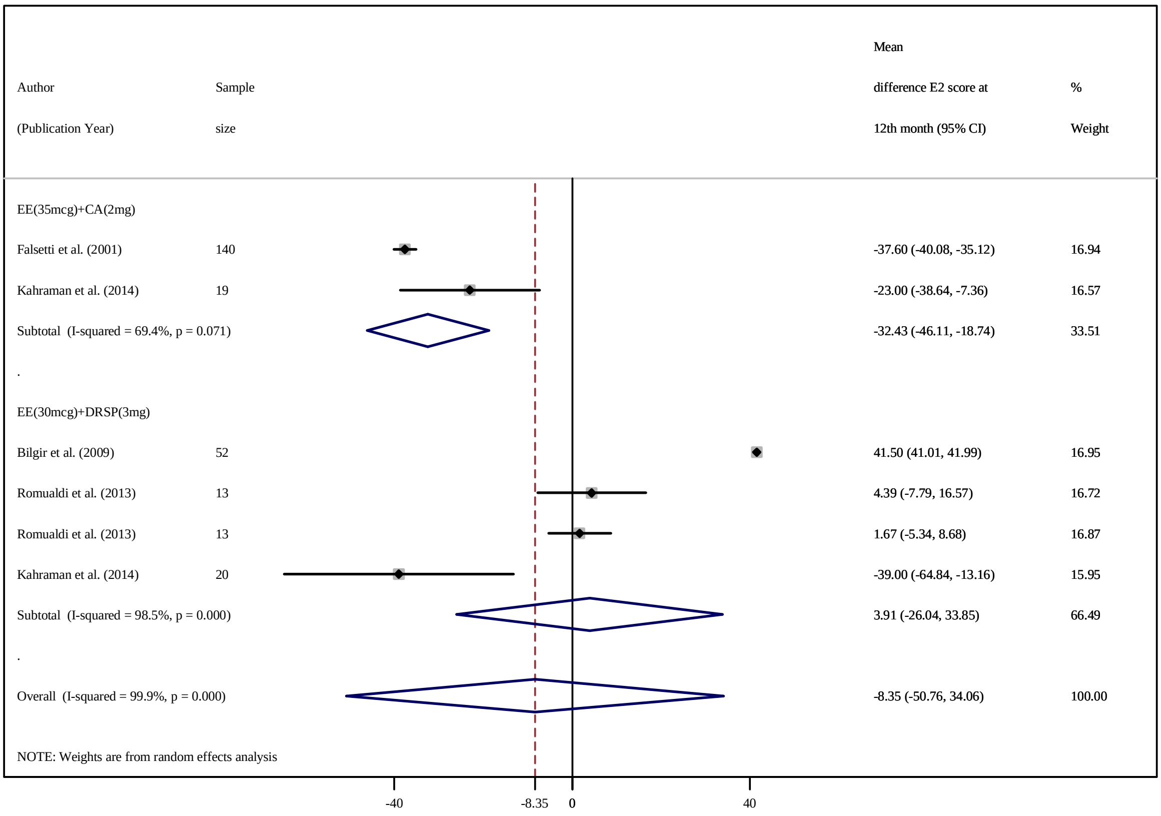

Figure 12. Forest plot of COCs effects on TT after 3 months of treatment.

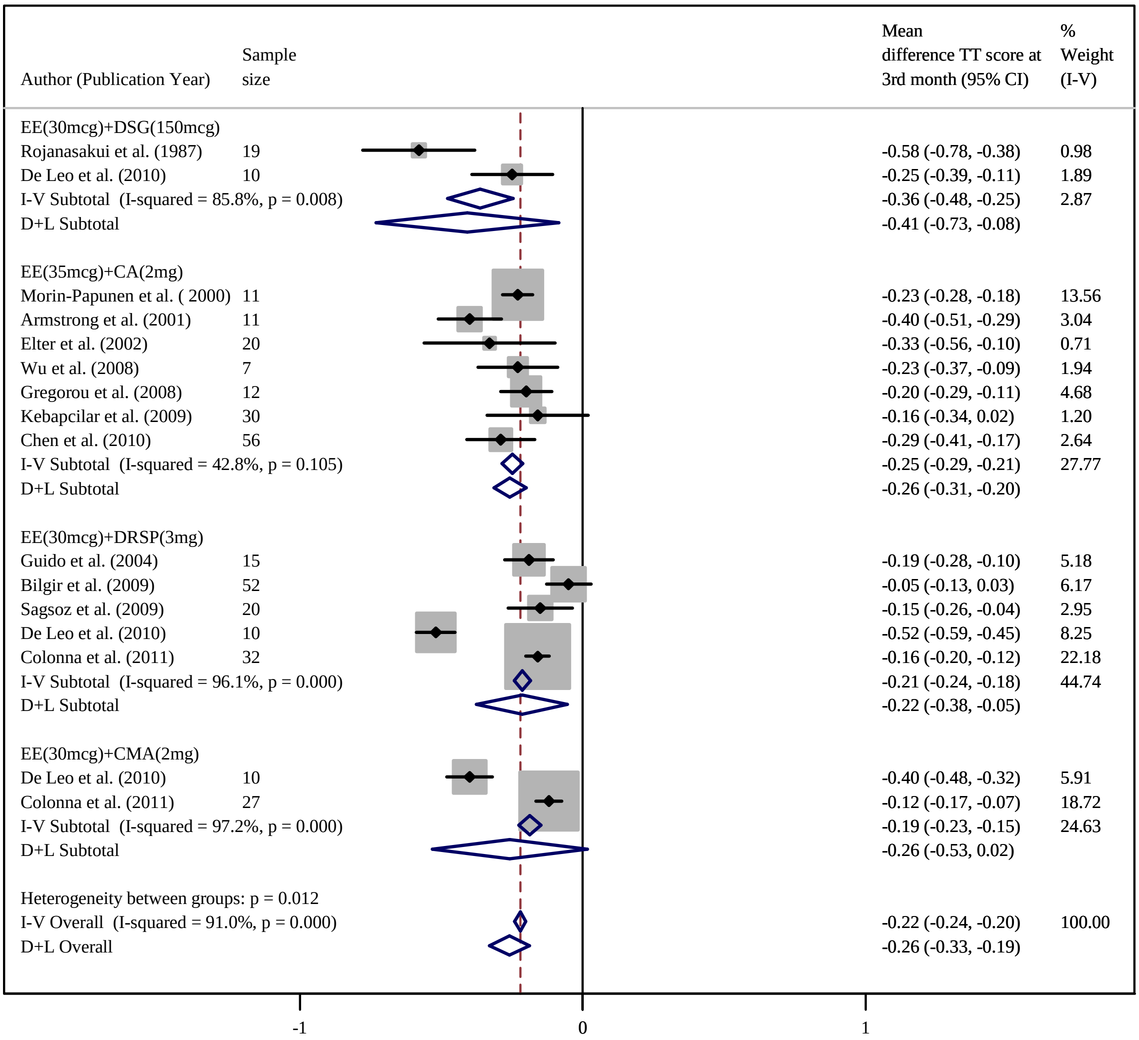

Figure 13. Forest plot of COCs effects on TT after 6 months of treatment.

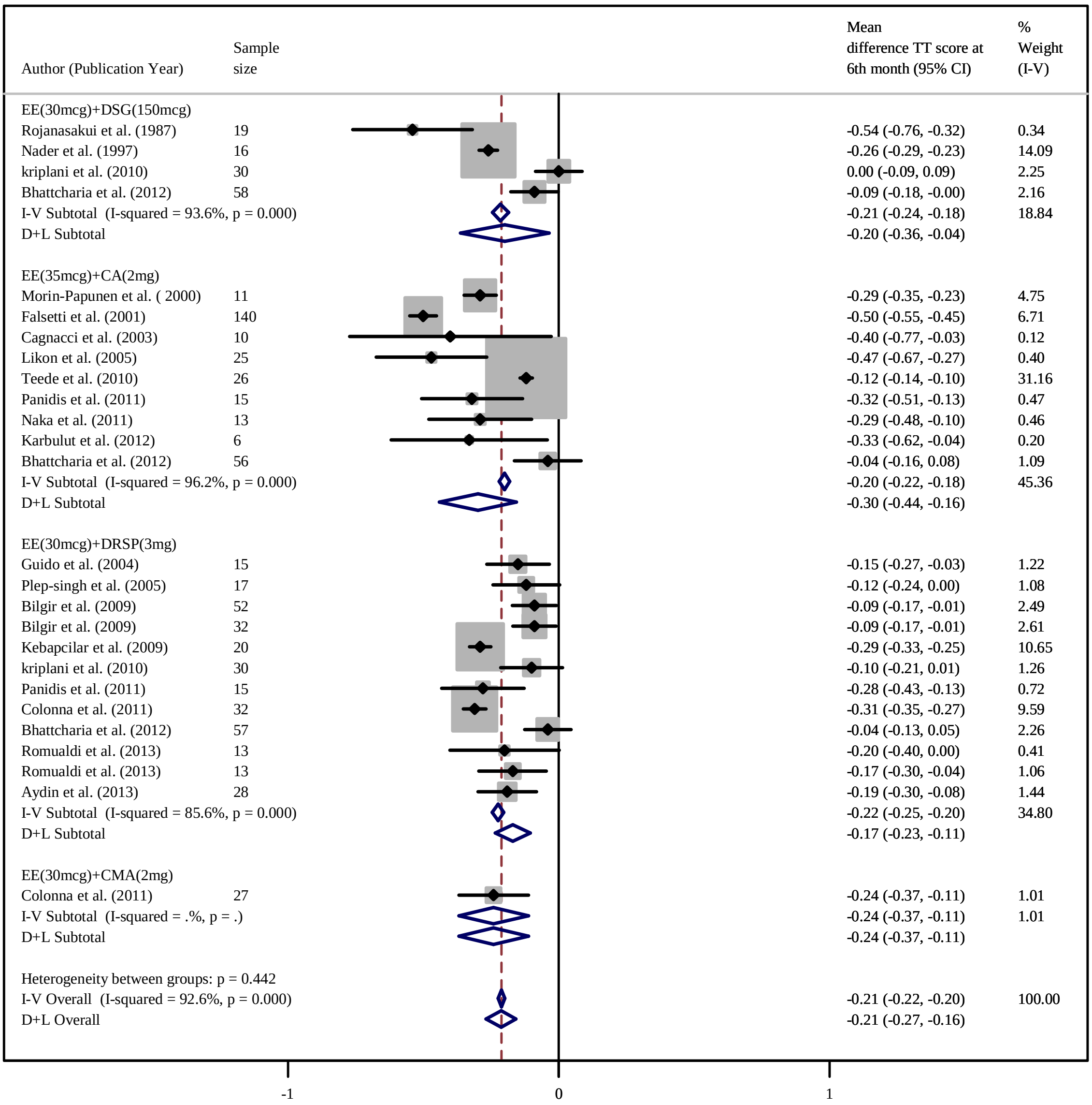

Figure 14. Forest plot of COCs effects on TT after 12 months of treatment.

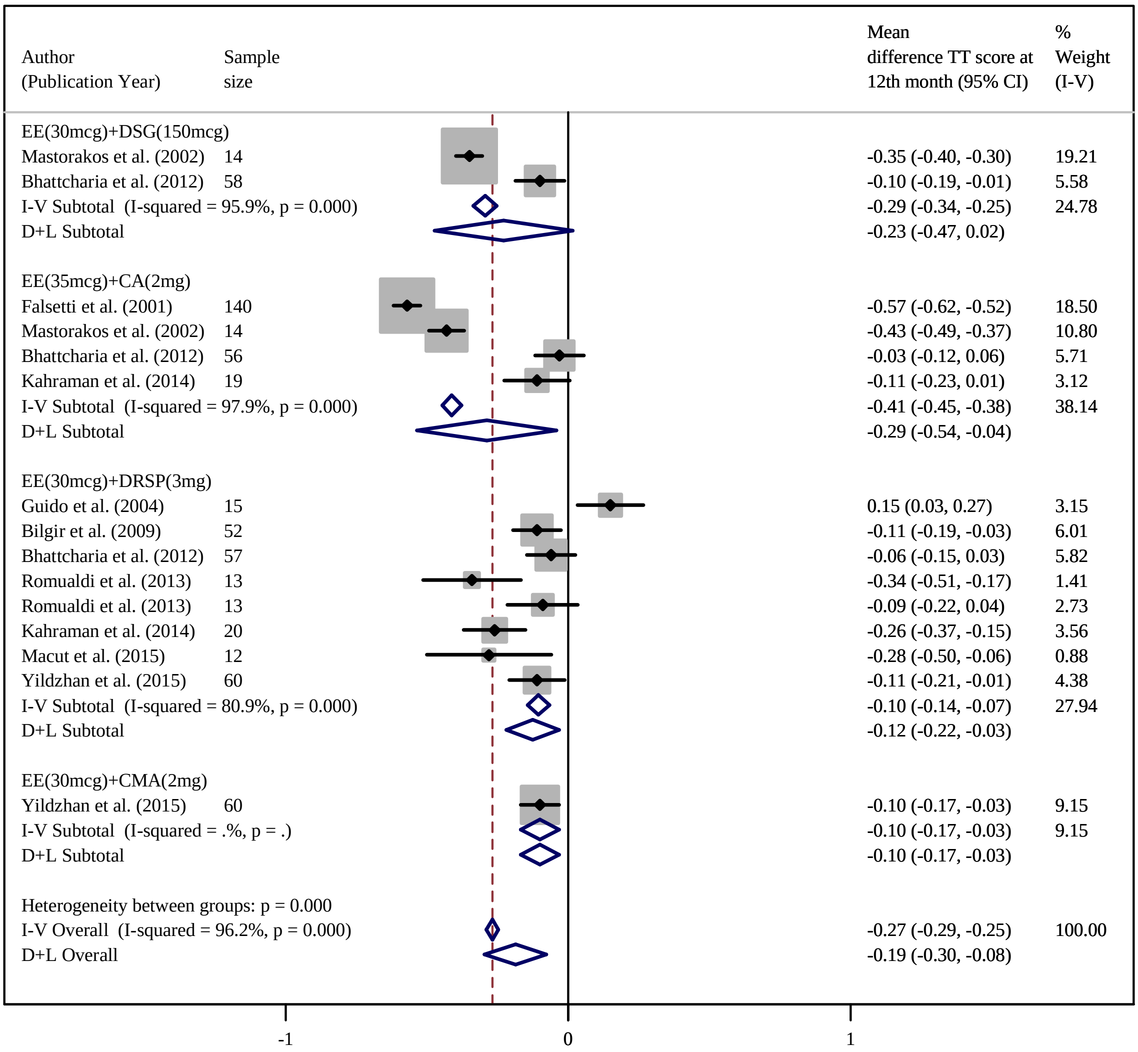

Figure 15. Forest plot of COCs effects on SHBG after 3 months of treatment.

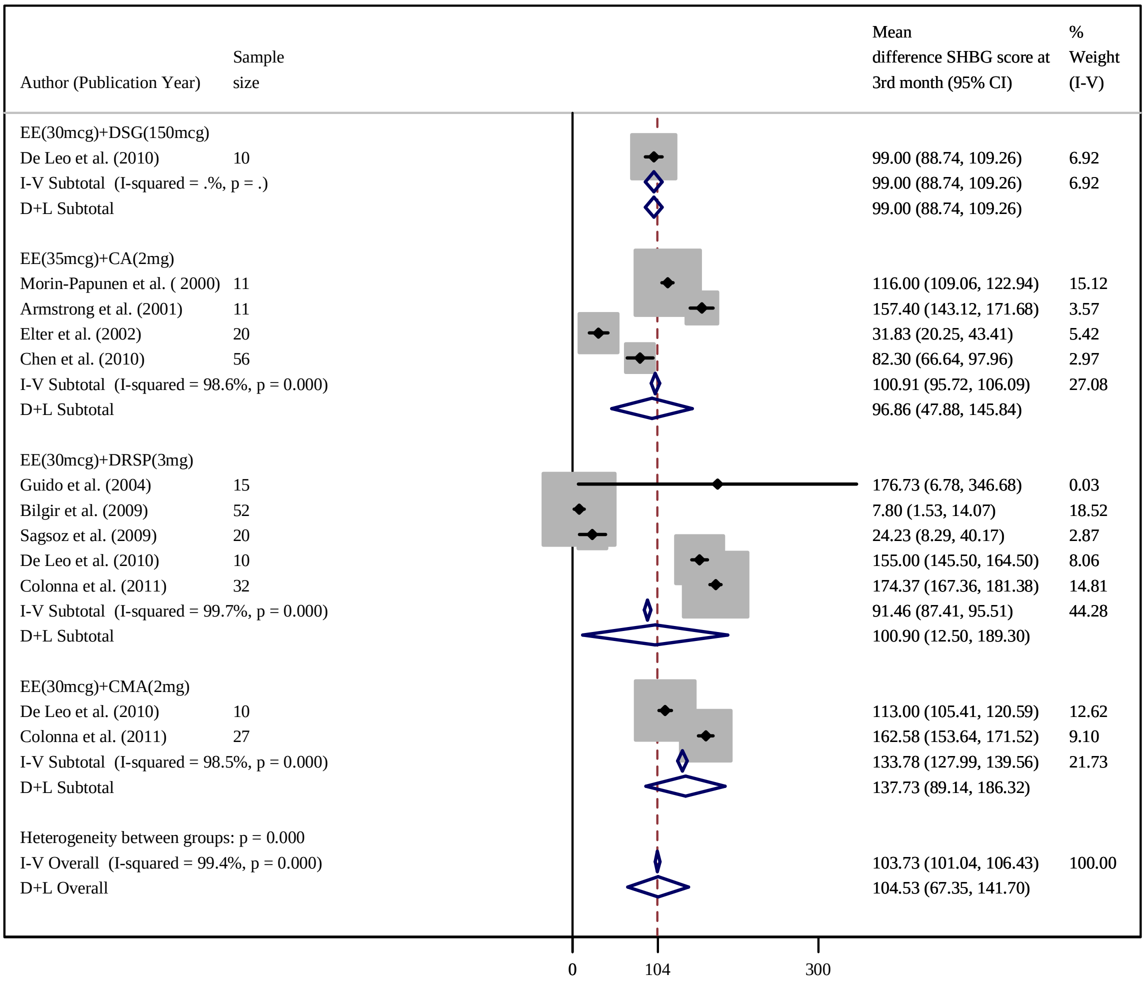

Figure 16. Forest plot of COCs effects on SHBG after 6 months of treatment.

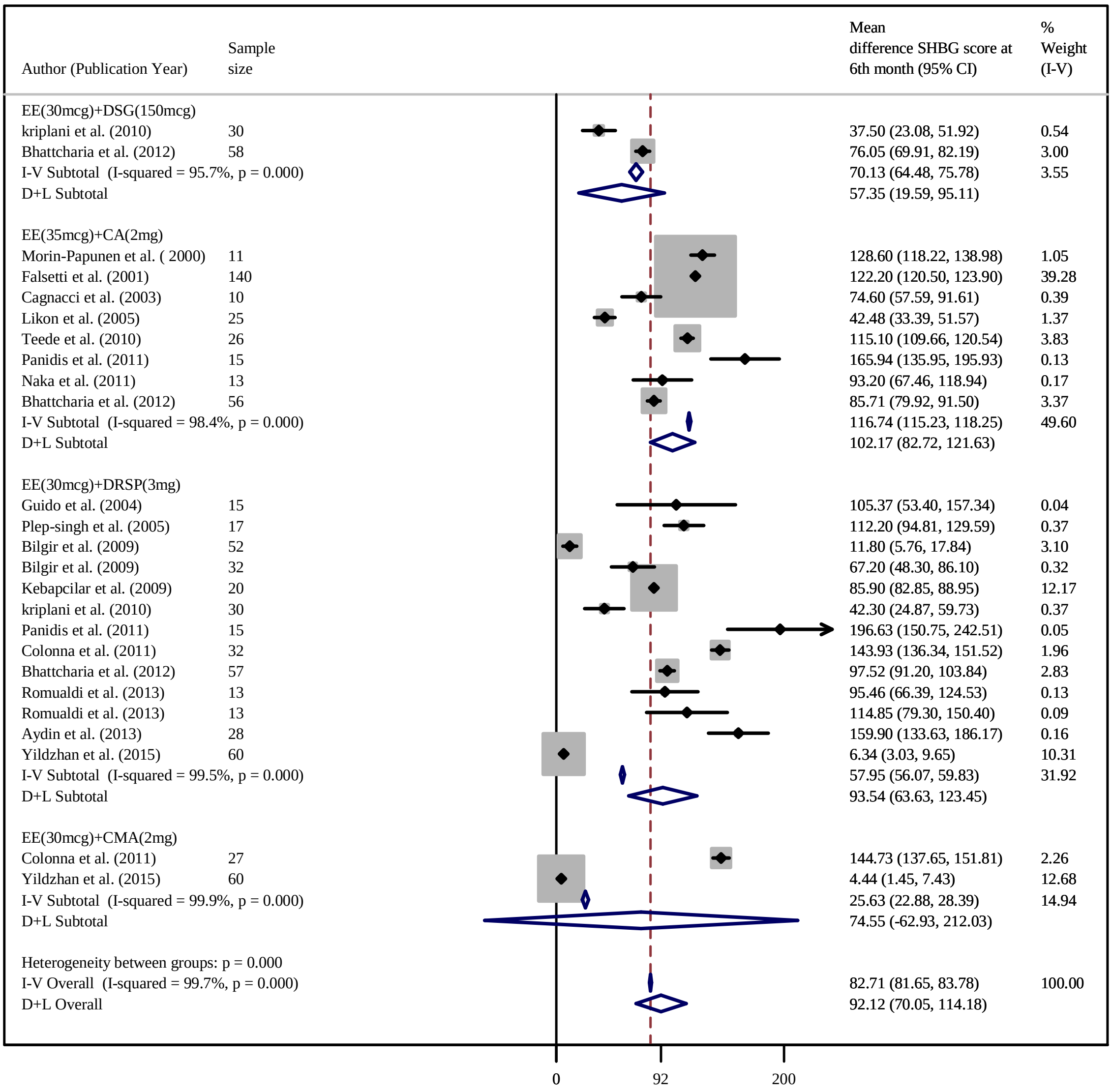

Figure 17. Forest plot of COCs effects on SHBG after 12 months of treatment.

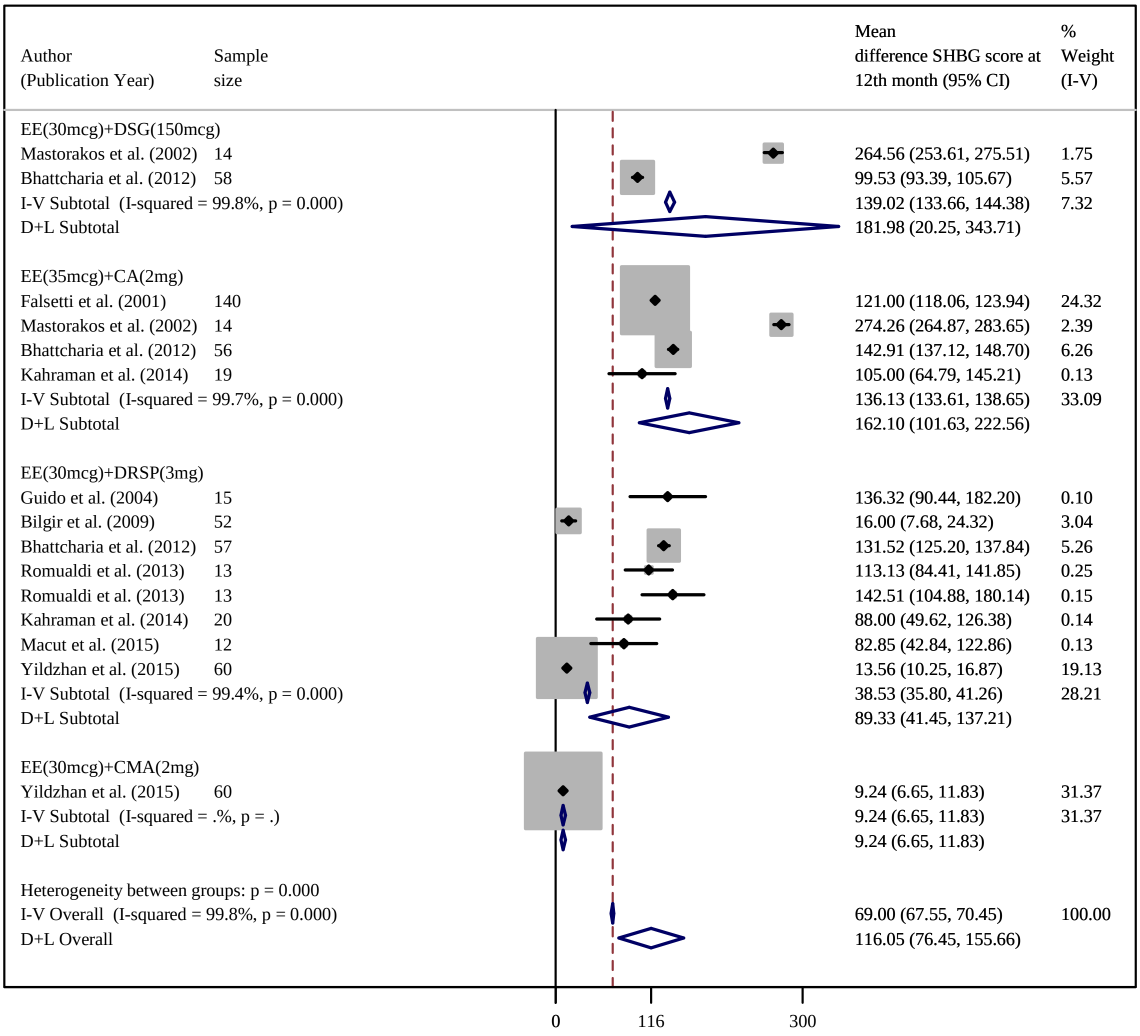

Supplement: Multimedia Appendix 5 [file resprot_v7i4e113_app5.pdf]
