# Supplementary material for: Comparing the Effects of Combined Oral Contraceptives Containing Progestins With Low Androgenic and Antiandrogenic Activities on the Hypothalamic-Pituitary-Gonadal Axis in Patients With Polycystic Ovary Syndrome: Systematic Review and Meta-Analysis
Source: JMIR Res Protoc. 2018 Apr 25;7(4):e113. doi: 10.2196/resprot.9024 (PMC5943622; doi:10.2196/resprot.9024)

Multimedia Appendix 6: (Funnel plots of publication bias and related corrections)

Figure 1. Publication bias on FSH after 6 months of treatment.

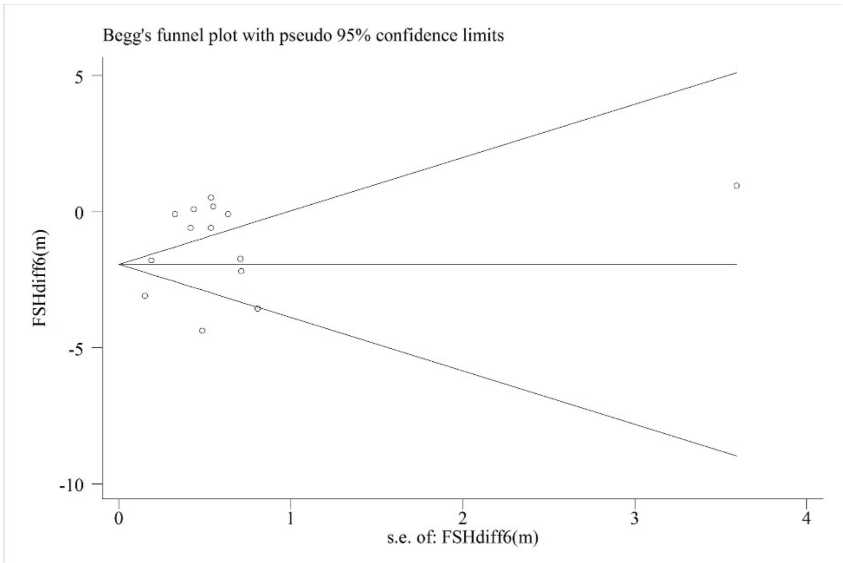

FSH diff after 6 months (P=0.011)

Before correction:

(MD= -1.30; 95% CI: -2.14, -0.46)

After correction:

(MD = -1.33; 95% CI: -2.16, -0. 49)

Figure 2. Correction of publication bias on FSH after 6 months of treatment.

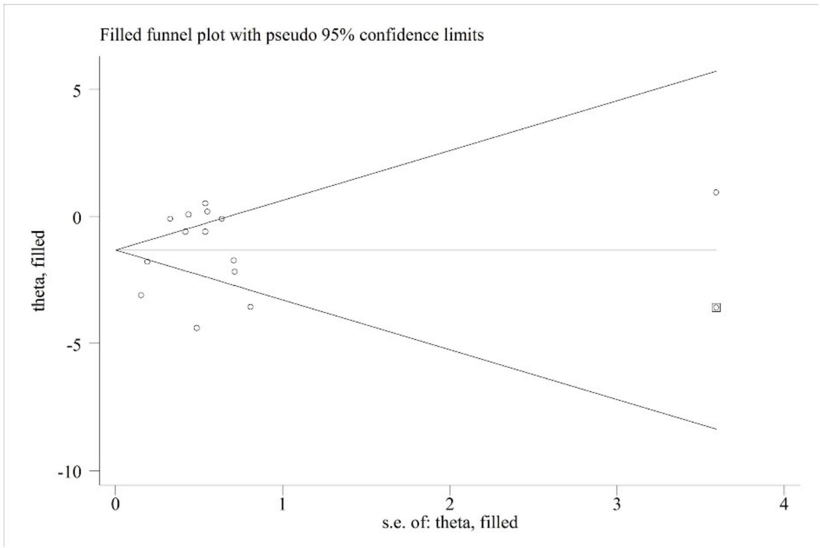

Figure 3. Publication bias on E2 after 6 months of treatment.

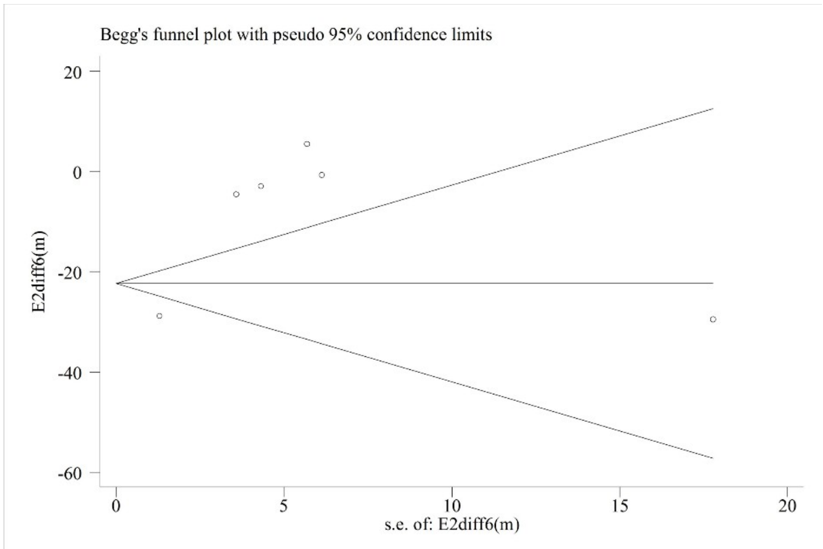

FSH diff after 6 months (P=0.011)

Before correction:

(MD = -8.96; 95% CI: -24.16, 6.24)

After correction:

(MD = -8.96; 95% CI: -24.16, 6.24)

Figure 4. Correction of publication bias on E2 after 6 months of treatment.

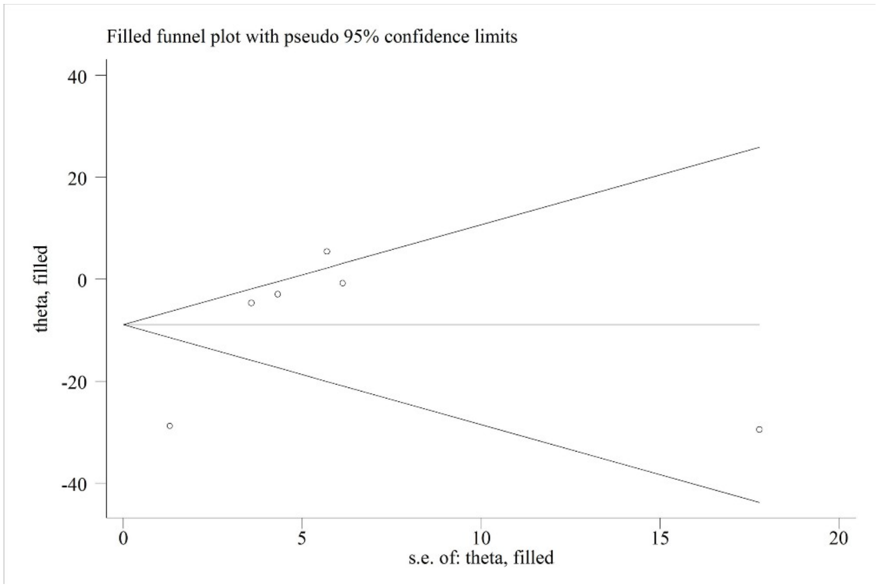

Supplement: Multimedia Appendix 6 [file resprot_v7i4e113_app6.pdf]
