# Supplementary material for: Comparing the Effects of Combined Oral Contraceptives Containing Progestins With Low Androgenic and Antiandrogenic Activities on the Hypothalamic-Pituitary-Gonadal Axis in Patients With Polycystic Ovary Syndrome: Systematic Review and Meta-Analysis
Source: JMIR Res Protoc. 2018 Apr 25;7(4):e113. doi: 10.2196/resprot.9024 (PMC5943622; doi:10.2196/resprot.9024)
Supplement: Multimedia Appendix 7 [file resprot_v7i4e113_app7.pdf]

The results of sensitivity analysis:

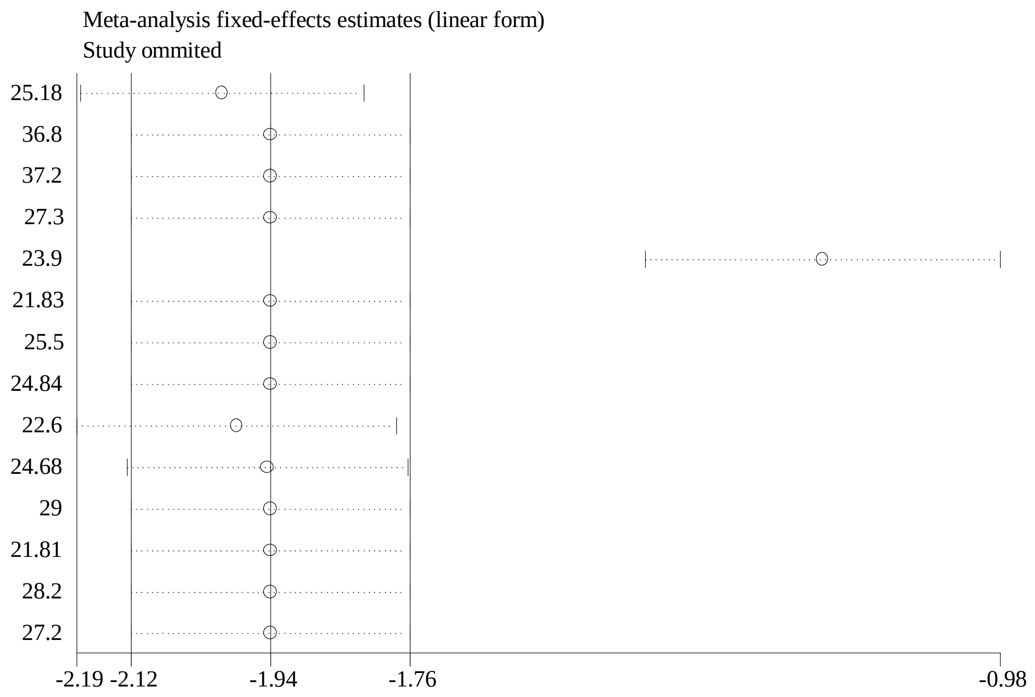

Figure 1. Sensitivity analysis for assessing the effect of BMI on FSH difference at 6th month from baseline level

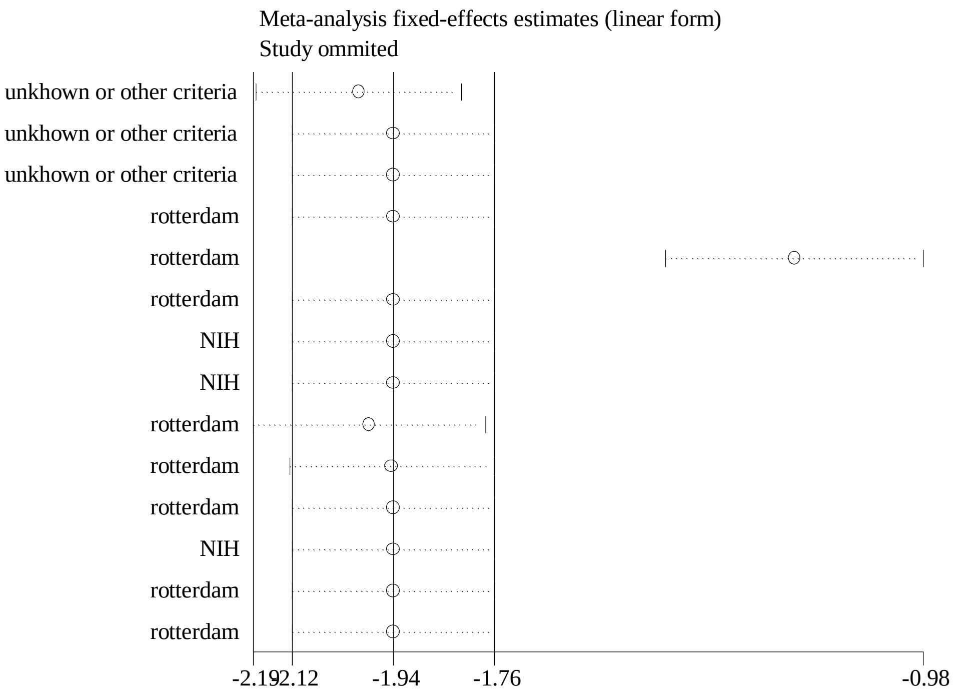

Figure 2. Sensitivity analysis for assessing the effect of diagnostic criteria of PCOS on FSH difference at 6th month from baseline level

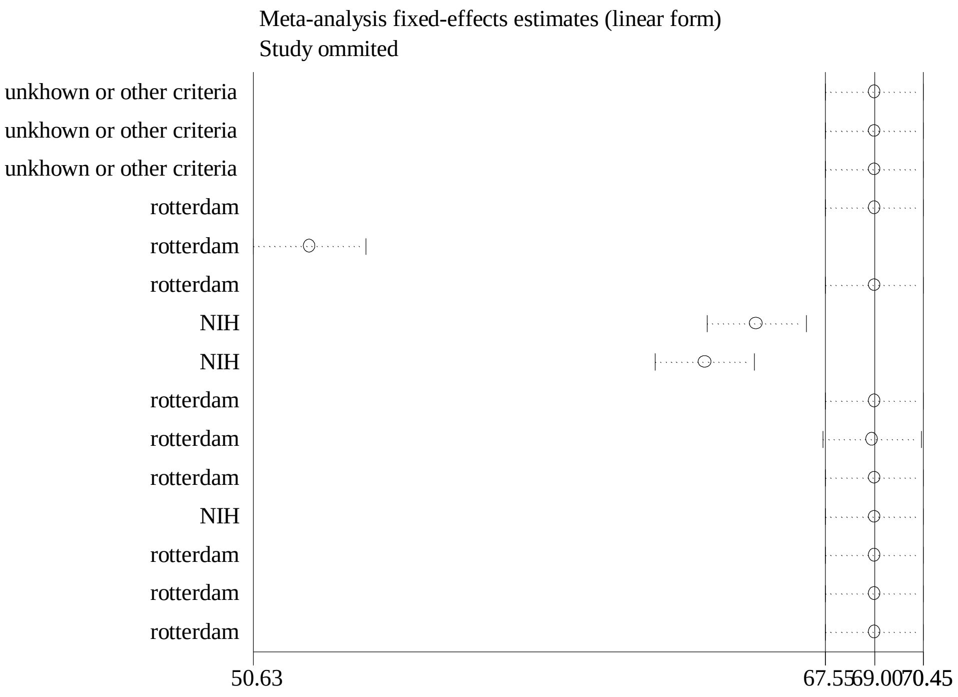

Figure 3. Sensitivity analysis for assessing the effect of diagnostic criteria of PCO on SHBG difference at 12th month from baseline level

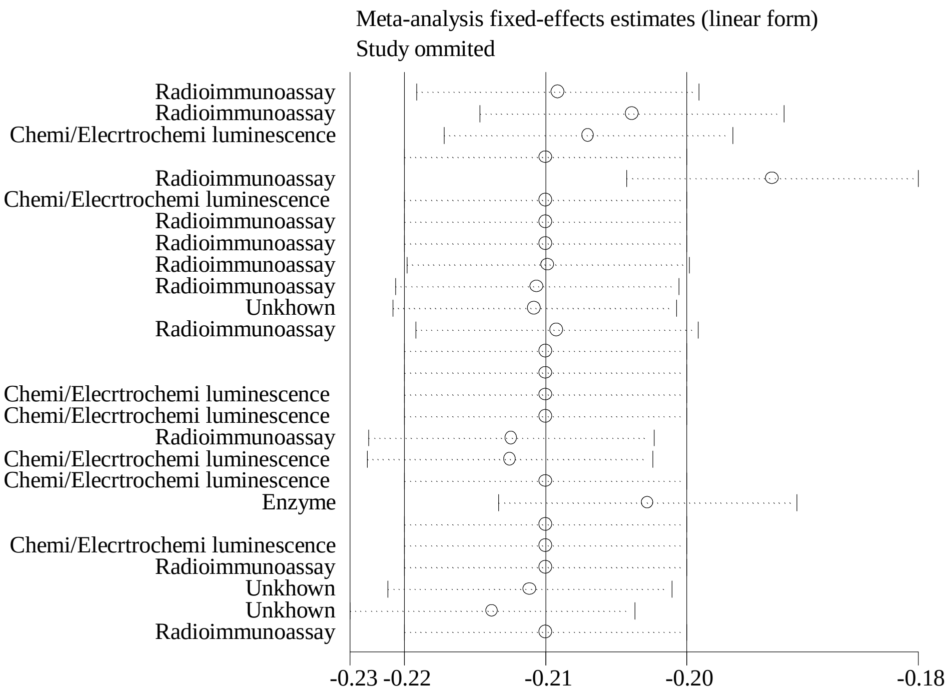

Figure 4. Sensitivity analysis for assessing the effect of method of assay for total testosterone on its difference at 6th month from baseline level

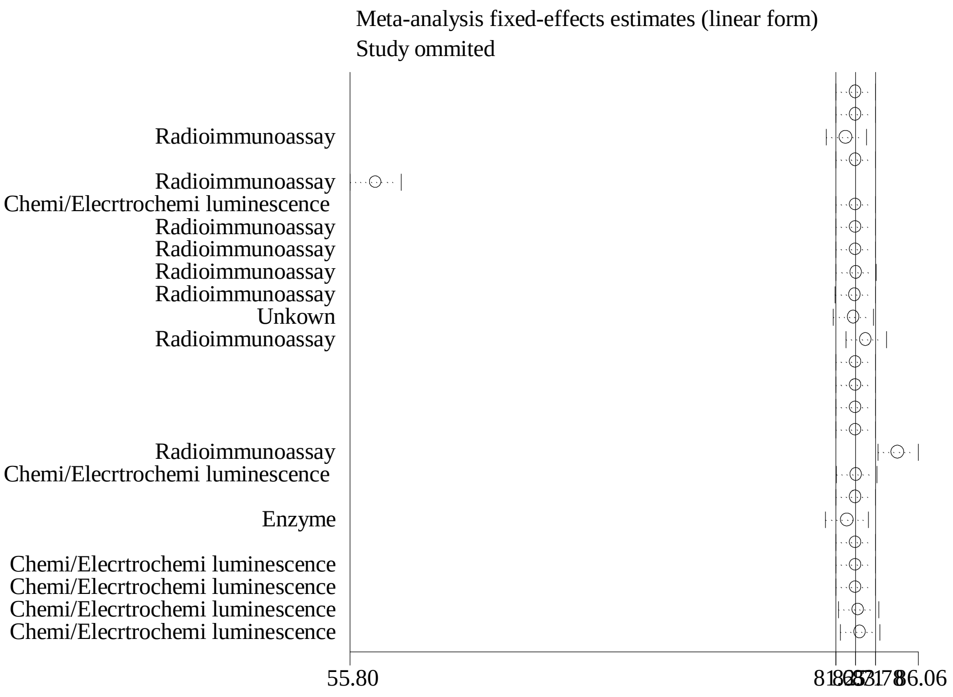

Figure 5. Sensitivity analysis for assessing the effect of method of assay for SHBG on its difference at 6th month from baseline level
