# Supplementary figures and images for: Comparing the Effects of Combined Oral Contraceptives Containing Progestins With Low Androgenic and Antiandrogenic Activities on the Hypothalamic-Pituitary-Gonadal Axis in Patients With Polycystic Ovary Syndrome: Systematic Review and Meta-Analysis
Source: JMIR Res Protoc. 2018 Apr 25;7(4):e113. doi: 10.2196/resprot.9024 (PMC5943622; doi:10.2196/resprot.9024)

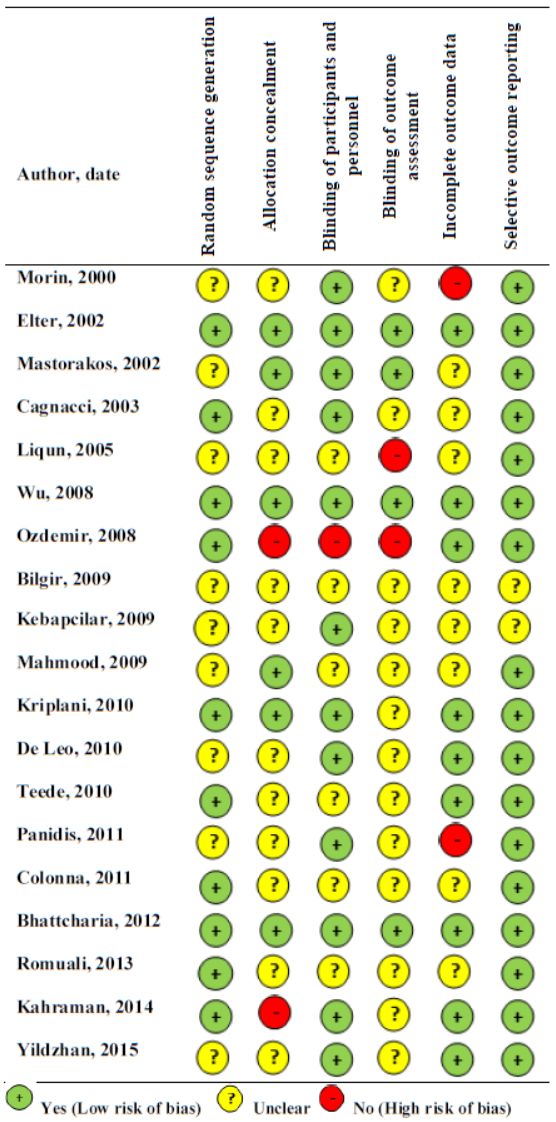

Supplement: Multimedia Appendix 8 [file resprot_v7i4e113_app8.jpg]

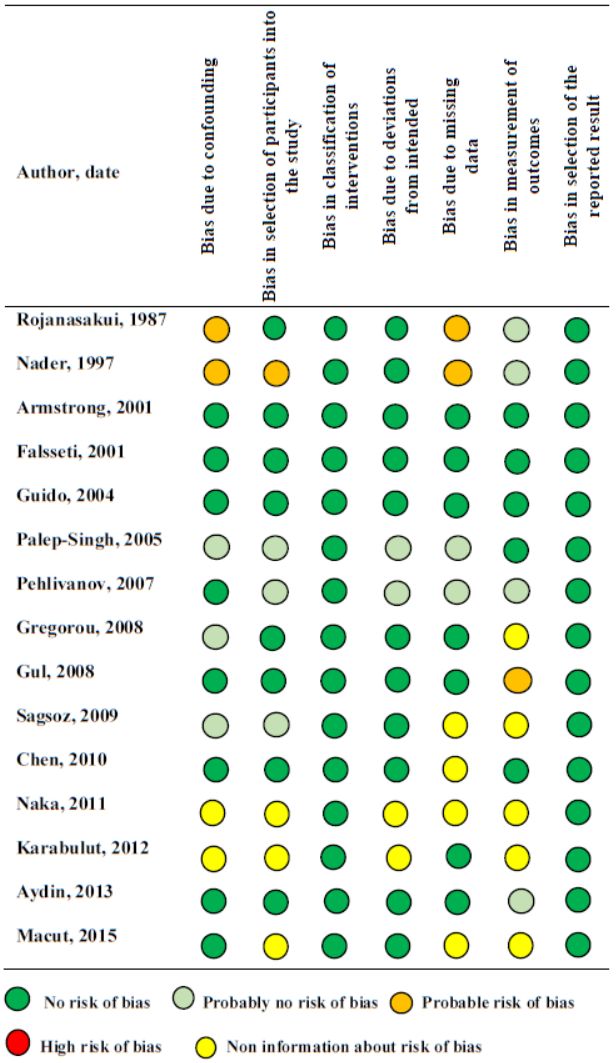

Supplement: Multimedia Appendix 9 [file resprot_v7i4e113_app9.jpg]
